# Novel miRNA-31 and miRNA-200a-Mediated Regulation of Retinoblastoma Proliferation

**DOI:** 10.1371/journal.pone.0138366

**Published:** 2015-09-17

**Authors:** Vanessa Montoya, Hanli Fan, Paul J. Bryar, Joanna L. Weinstein, Marilyn B. Mets, Gang Feng, Joshua Martin, Alissa Martin, Hongmei Jiang, Nikia A. Laurie

**Affiliations:** 1 Human Molecular Genetics Program, Lurie Children’s Research Center, Chicago, Illinois, 60611, United States of America; 2 Department of Pediatrics, Northwestern University Feinberg School of Medicine, Chicago, Illinois, 60611, United States of America; 3 Department of Ophthalmology, Northwestern University Feinberg School of Medicine, Chicago, Illinois, 60611, United States of America; 4 Department of Pathology, Northwestern University Feinberg School of Medicine, Chicago, Illinois, 60611, United States of America; 5 Division of Hematology, Oncology, and Stem Cell Transplantation, Ann & Robert H. Lurie Children’s Hospital of Chicago, Chicago, Illinois, 60611, United States of America; 6 Division of Ophthalmology, Ann & Robert H. Lurie Children’s Hospital of Chicago, Chicago, Illinois, 60611, United States of America; 7 Clinical & Translational Sciences Institute, Northwestern University Feinberg School of Medicine, Chicago, Illinois, 60611, United States of America; 8 Department of Statistics, Weinberg College of Arts & Sciences, Northwestern University, Evanston, Illinois, 60208, United States of America; Indiana University School of Medicine, UNITED STATES

## Abstract

Retinoblastoma is the most common intraocular tumor in children. Current management includes broad-based treatments such as chemotherapy, enucleation, laser therapy, or cryotherapy. However, therapies that target specific pathways important for retinoblastoma progression could provide valuable alternatives for treatment. MicroRNAs are short, noncoding RNA transcripts that can regulate the expression of target genes, and their aberrant expression often facilitates disease. The identification of post-transcriptional events that occur after the initiating genetic lesions could further define the rapidly aggressive growth displayed by retinoblastoma tumors. In this study, we used two phenotypically different retinoblastoma cell lines to elucidate the roles of miRNA-31 and miRNA-200a in tumor proliferation. Our approach confirmed that miRNAs-31 and -200a expression is significantly reduced in human retinoblastomas. Moreover, overexpression of these two miRNAs restricts the expansion of a highly proliferative cell line (Y79), but does not restrict the growth rate of a less aggressive cell line (Weri1). Gene expression profiling of miRNA-31 and/or miRNA-200a-overexpressing cells identified differentially expressed mRNAs associated with the divergent response of the two cell lines. This work has the potential to enhance the development of targeted therapeutic approaches for retinoblastoma and improve the efficacy of treatment.

## Introduction

Retinoblastoma is the most common intraocular tumor in children worldwide. Although it is highly curable when detected early, the mortality rate is as high as 70% in less-developed countries [1]. This poor outcome is largely due to late detection likely related to socioeconomic disparities. Additionally, 23% of eyes requiring enucleation in the United States display high-risk histopathologic features [2]. These features include invasion of the optic nerve beyond the lamina cribrosa, which is a known metastatic risk factor [3]. Advanced stage retinoblastoma is challenging to treat because retinoblastomas can rapidly fill the eye, invade the optic nerve, and eventually spread to the central nervous system, thus becoming fatal. Importantly, a favorable outcome is dependent upon an early diagnosis, in addition to the success of broad-based treatments such as chemotherapies, enucleation, laser therapy, or cryotherapy [4]. Children diagnosed with retinoblastoma tend to present before 2 years of age with one or both eyes affected [5]. Most retinoblastomas initiate from a bi-allelic loss of the *RB1* gene [6], which encodes a critical regulator (pRb) of the cell cycle. Retinoblastoma progression involves inactivating pathways such as, Arf–MDM2/MDMX–p53 [7], while maintaining a relatively low mutation rate [8]. The identification of genetic changes that occur after the initiating genetic lesion is necessary to further define the aggressive proliferative ability of retinoblastoma tumors.

MicroRNAs (miRNAs, miRs) are small, non-coding RNA transcripts that can simultaneously repress multiple genes via perfect or imperfect base pairing (see review [9]). MiRNA profiling of multiple cancer types indicates that tumor tissues can be distinguished from normal tissues of the same origin [10]. Further, monoallelic loss of an essential endonuclease (*Dicer1*) in canonical miRNA biosynthesis promotes retinoblastoma tumorigenesis [11]. Therefore, the identification of miRNAs is important for elucidating targets that are necessary for retinoblastoma progression.

In a profile of 29 human retinoblastomas, miRNA-31 was first reported to be downregulated as compared to 6 normal human retinas [12]. This is a notable observation because miRNA-31 exhibits tumor suppressive effects in cancer models, including the ovary [13], the pancreas [14], and the brain [15]. For retinoblastoma studies, the potential for miRNA-31 and/or -200a to restrict the growth of this rapidly growing tumor and, by consequence reduce its likelihood to invade ocular structures, is critical because evidence of optic nerve invasion is an important feature with adverse prognostic value for the patient [3]. A second miRNA with the potential to inhibit retinoblastoma proliferation is miRNA-200a, which we identified in a miRNA profiling study of 12 retinoblastomas [16]. MiRNA-200a belongs to a family of miRNAs that are significant regulators of cancer metastasis [17], neuronal differentiation and cell cycle exit [18]. As a suppressor of the Wnt/β-Catenin pathway in meningiomas [19], miR-200a is of great interest because Wnt activation is associated with expansion of a highly malignant cancer stem cell-like population within retinoblastoma cell cultures [20]. MiRNAs-31 and -200a thus potentially represent novel regulators of retinoblastoma progression.

We sought to elucidate the roles of miRNA-31 and miRNA-200a in retinoblastoma proliferation and apoptosis. For this study, we used two phenotypically different cell lines (Y79 [21] and Weri1 [22]), one highly proliferative and the other less aggressive, that are widely used for retinoblastoma studies. To this effect, we would be able to better describe the extent to which expression of miRNAs-31 and/or -200a may be correlated with a more aggressive type of retinoblastoma. Furthermore, it would allow us to determine if miRNAs-31 or -200a exhibit differential functions in culture. Our findings collectively suggest that contextually dependent loss of miRNA-31 and miRNA-200a expression promotes retinoblastoma progression through selective downregulation of targets associated with rapid proliferation.

## Materials and Methods

### Clinical samples

Twenty-six human tissue samples were used for this study, which included five normal pediatric retinas and twenty-one retinoblastomas. The five normal pediatric retinas were dissected from whole globes obtained from The National Disease Research Interchange (www.ndri.org) and Eversight (eversightvision.org). The mean age of our cohort of normal retinas was 9.8 months (± 8.95). The retinoblastomas were formalin-fixed paraffin-embedded (FFPE) products of enucleation obtained from Ann & Robert H. Lurie Children's Hospital of Chicago and Northwestern University Feinberg School of Medicine. Prior to miRNA analysis, all retinoblastomas were microscopically analyzed and macro-dissected, as we previously described [16].

### Ethics Statement

Human tissues were obtained under human subject research protocols approved by the Institutional Review Boards at Ann & Robert H. Lurie Children's Hospital of Chicago (IRB Project Number 2010–14211) and Northwestern University Feinberg School of Medicine (IRB Project Number STU00035305). The Institutional Review Board waived the requirement of obtaining informed consent for this study in accordance with 45 CFR 46.116(d).

### Cell culture

Human retinoblastoma cell lines Y79 and WERI-Rb1 (Weri1), obtained from ATCC^®^ (Catalog# HTB-18 and HTB-169, respectively), were maintained in RPMI 1640 (Corning) containing 10% FBS (Hyclone) and 1% PSG (penicillin, streptomycin, glutamine), and incubated at 37°C with 5% CO_2_.

### Quantitative RT-PCR analysis

Validations of miRNA-31 and miRNA-200a were performed using TaqMan® MicroRNA Individual assays (Applied Biosystems) for real-time PCR. Total RNA (10ng) from control pediatric retinas, retinoblastomas, and retinoblastoma cell lines was reverse transcribed using the MicroRNA Reverse Transcription kit (Applied Biosystems) and analyzed using MicroRNA Individual Assays according to the manufacturer’s instructions. For miRNA qRT-PCR with twenty-one retinoblastomas (21 individual patients) as compared to five normal pediatric retinas (five individual patients), samples were treated as independent biological replicates. The experiment was performed in duplicate to assess the mean negative delta Ct (-ΔCt). For miRNA qRT-PCR in two human retinoblastoma cell lines as compared to three (individual) retinas, experiments were completed using triplicate samples of each cell line and normal retina, and performed in duplicate to assess the mean RQ (fold change) and standard deviation. All microRNA expression was normalized to endogenous control *RNU48*. For gene repression validations following miRNA overexpression, reverse transcription of total RNA (1000ng) was completed using the High-Capacity RNA-to-cDNA^TM^ Kit (Applied Biosystems). These samples represent RNA isolated at 48 hours post-transfection from two independent experiments that were completed in triplicate using human TaqMan® Gene Expression Assays (Life Technologies), normalized to *GAPDH*. Reverse transcription of total RNA (50ng, harvested 48 hours post-transfection) to confirm depletion target mRNAs (*DLL3*, *ACOT7*), following two independent siRNA transfections in retinoblastoma cells (Y79, Weri1), in addition to quantification of endogenous *DLL3* and *ACOT7* in retinoblastoma cell lines and three normal, pediatric retinas, was completed using the High-Capacity RNA-to-cDNA^TM^ Kit (Applied Biosystems). This was followed with human TaqMan® Gene Expression Assays (Life Technologies) for real-time PCR, normalized to GAPDH. RQ was calculated using the 2^−Δ(ΔCt)^ method where RQ or fold change is equal to 2^−((Mean ΔCt Target) − (Mean ΔCt Calibrator))^.

### *In silico* analysis to identify pathways involving miRNA-31 and miRNA-200a regulation

GOmir [23] was used to identify miRNA-31 and miRNA-200a predicted target genes. Pathway analysis was completed using MetaCore^TM^ (Thomson Reuters) to calculate the statistical significance of gene enrichment within their human-curated database of signaling pathways and networks.

### mirVana^TM^ mimic transfections

Human retinoblastoma cell lines (Y79 and Weri1) were reverse transfected using RNAiMAX (Invitrogen) in triplicate with 30 picomoles of a negative mirVana^TM^ mimic (Ambion, Catalog #: 4464058), or, miRNA-31 mimic (Ambion, Catalog #: 4464066, ID: MC11465), or miRNA-200a (Ambion, Catalog #: 4464066, ID: MC10991), or, “Mix” where the “Mix” is a 1:1 combination of miRNA-31 mimic and miRNA-200a mimic per 5.0E4 cells/well in a 24-well plate. Human retinoblastoma cells were reverse transfected according to the manufacturer’s protocol, as we previously described [24].

### Silencer® siRNAs transfections

Human retinoblastoma cell lines (Y79 and Weri1) were reverse transfected in triplicate with 30 picomoles of a negative silencer siRNA #1 (Ambion, Catalog #: AM4611), silencer DLL3 (Ambion, Catalog #: AM16708, ID: 136952 (siRNA #1), 136953 (siRNA #2), silencer ACOT7 (Ambion, Catalog #: AM16708, ID: 19438 (siRNA #1), ID: 121122 (siRNA #2)), silencer STK40 (Ambion, Catalog #: AM16708, ID: 103725), or silencer PPP6C (Ambion, Catalog #: AM16708, ID: 104527), per 5.0E4 cells/well in a 24-well plate. Human retinoblastoma cells were reverse transfected according to the manufacturer’s protocol and as previously described [24].

### mirVana^TM^ miRNA inhibitor transfections

Human retinoblastoma cell lines (Y79 and Weri1) were reverse transfected using RNAiMAX (Invitrogen) in triplicate with 30 picomoles of negative mirVana^TM^ mimic inhibitor (Ambion, Catalog #:4464076), miRNA-31-5p inhibitor (Ambion, Catalog #:4464084, ID: MH11465), miRNA-200a-3p inhibitor (Ambion, Catalog #:4464084, ID:MH10991), or “Mix”. “Mix” is a 1:1 combination of miRNA-31 and miRNA-200a inhibitor per 5.0E4 cells/well in a 24-well plate. Human retinoblastoma cells were reverse transfected according to the manufacturer’s protocol, as we previously described [24].

### Luciferase assay

A firefly luciferase encoding plasmid vector, pMirTarget, containing the 3’UTR of *ACOT7* (*ACOT7*, NCBI Reference Sequence: NM_007274.3), referred to as pMir-ACOT7, was purchased from Origene (custom order). A second luciferase-encoding vector (pMir-mut) contains three changed nucleotides within the putative miR-200a binding site of the 3’UTR of *ACOT7* (Origene, custom order). Forty-eight hours after reverse transfection of the pMir plasmid (1143ng), cells were harvested. Reverse transfection was conducted using Lipofectamine 2000 (Invitrogen), in accordance with manufacturer’s instructions, and in the presence of 30 pmol of a negative or miR-200a mimic, in addition to a renilla encoding plasmid (285ng, Life Technologies) for normalization purposes. Firefly and renilla luciferase activities were measured using the dual-luciferase reporter assay system (Promega).

### RNA isolation

RNA from normal human retinal tissue, human cell lines, and transfected cell lines was isolated using TriZol reagent (Invitrogen). RNA isolation was completed according to manufacturer’s protocol. RNA isolation from FFPE retinoblastoma tissues was performed using RecoverAll^TM^ Nucleic Acid Isolation (Ambion) according to manufacturer’s protocol.

### Cell proliferation and apoptosis assays

Cell proliferation was determined using the Guava ViaCount® reagent (Millipore) according to manufacturer’s protocol. Data represents the mean percent of total cells/mL of retinoblastoma cells from three independent experiments with triplicate samples, unless otherwise noted. Total percent apoptosis was determined using the Guava Nexin^TM^ Annexin V stain (Millipore), according to manufacturer’s protocol. Data represents the mean value of the sum proportion of cells in early and late stage apoptosis from three independent experiments with triplicate samples.

### Gene expression microarray and bioinformatic analysis

Gene expression analysis was performed using the Illumina Human HT-12 BeadChip (Illumina), which provides coverage of more than 47,000 genes and expressed sequence tags. cRNA from cells treated with miRNA mimics was generated using a commercial kit (Illumina® TotalPrep™-96 RNA Amplification Kit, Ambion). Labeled RNA was next hybridized to Human HT-12 arrays. Raw signal intensities of each probe were obtained using data analysis software (BeadStudio, Illumina) and imported to the lumi package of Bioconductor for data analysis. Before transformation and normalization [25–27], A/P call detection was performed based on detection p-value. 23844 out of 47314 probes with p-value less than 0.01 were considered as valid signals. Downregulated genes were identified using an Analysis of Variance (ANOVA) model with empirical Bayesian variance estimation [28]. Genes were identified as being downregulated on the basis of statistical significance (raw p-value < 0.01 and false discovery rate adjusted p-value < 0.05), and 1.5-fold change (down) in expression level for the comparisons between the specified miRNA overexpressing samples and controls (negative miRNA mimic). RNA used for this study was isolated from cells at 48 hours post-transfection from three independent experiments. RNA was bioanalyzed, labeled, and hybridized to the array by the University of Chicago Genomics Facility. Raw data was collected by the University of Chicago Genomics Facility and analyzed by our bioinformatics specialist, Gang Feng, PhD (Northwestern University).

### Statistical analyses

Statistical analyses were performed using R 3.0.2 and SAS Studio 3.2. The miRNA expression as measured by negative Delta Ct (-ΔCt) was compared between 21 individual retinoblastoma tumors and 5 pediatric controls using Wilcoxon rank sum test. One-way ANOVA, with Dunnett’s adjustment for multiple correction, was used to compare the miRNA expression measured by log10(RQ) in the two cell lines (Y79 and Weri1) and three individual pediatric controls. The same method was used to analyze cell proliferation, total apoptosis, and quantification of immunofluorescence, and to confirm miRNA overexpression (in log10 scale) following miRNA transfection and to confirm reduction of *DLL3* and *ACOT7* following siRNA transfection. Target mRNA expression, as measured in log10, in Y79 and Weri1 retinoblastoma cells after expressing miR-31, miR-200a, or “Mix” (miR-31/-200a), as compared to control treated cells were measured for 4 genes (*ACOT7*, *DLL3*, *PPP6C*, *STK40*). One-way ANOVA with Tukey’s method, was used to compare between Y79 and Weri1 for each combination of treatment and gene. One-way ANOVA with Dunnett’s adjustment was used to compare the relative quantification of *DLL3* and *ACOT7*, in log2, in Y79 and Weri1 cell lines versus three individual pediatric controls. Two-sample t-test with Satterthwaite method was used to analyze the luciferase data.

All tests were two-sided and the ones with p-values less than 0.05, after multiple testing correction when needed (such as Dunnett’s method), were considered statistically significant.

### Immunofluorescence staining and analysis

Briefly, Y79 cells (50,000–80,000) were allowed to adhere to poly-l-lysine coated slides and incubated for thirty-minutes in a humidified chamber. Adhered cells were fixed with 4% paraformaldehyde in PBS for twenty minutes at room temperature, followed by three washes. Endogenous peroxidases were quenched by incubating in 3% H_2_O_2_ in PBS for ten minutes, and then washed 3 times. Blocking buffer was added for one hour (1.35% normal goat serum, 3.33% of 3% BSA in 1.0mL PBS), and was followed by incubation with rabbit anti-human antibody (STK40, Abcam ab96290; DLL3, Acris AP51274PU-N; PPP6C, Abcam ab131335; or ACOT7, Abcam ab85151) overnight at 4°C, diluted at 1:400 in blocking buffer. Stained slides without added primary antibody were used as negative controls. Slides were washed three times and incubated with biotin-labeled goat-anti-rabbit secondary antibody (0.4% in blocking buffer) for forty-five minutes at room temperature using the Ultra-Sensitive ABC Rabbit IgG Staining Kit (Thermo Scientific Catalog #32054). Slides were washed three times. Avidin/streptavidin-conjugated with HRP in PBS was added to each slide and incubated for thirty minutes at room temperature. Slides were washed three times and treated with Tyramide Signal Amplification (TSA™) reagents for ten minutes at room temperature (0.2% TSA, 0.67% of 3% H_2_O_2,_ in 3.0mLs of PBS) to improve protein detection. Slides were mounted using mounting medium containing DAPI (Vector Laboratories). Unless noted otherwise, all steps were performed at room temperature in a humidified chamber, and washes with PBS (containing 0.05% Tween-20) were done between each step for 5 minutes. Data represent findings from two independent experiments with four independent measurements obtained in 8-bit grayscale. Cells used for this study were harvested 72 hours post-transfection.

### Western blot analysis

At 48 hours following miRNA transfection, cells were washed twice with PBS and resuspended in RIPA lysis buffer (Thermo Scientific) containing protease and phosphatase inhibitors (Thermo Scientific). Total protein was separated in 4–15% Mini-PROTEAN TGX Precast Protein Gels (Bio Rad) and transferred to nitrocellulose membranes. Blots were blocked in 5% albumin from bovine serum (Sigma) for 2 hours at room temperature, and incubated overnight at 4°C with primary antibody (DLL3, Acris AP51274PU-N; or ACOT7, Abcam ab85151) and diluted in blocking buffer (1:1000). Blots were incubated with anti-STK40 (Abcam ab96290) or anti-PPP6C (Abcam ab131335) diluted in blocking buffer (1:1000) for 1 hour at room temperature. After washes in TBS-T (0.001% tween-20), blots were incubated with horseradish peroxide-conjugated rabbit secondary antibody (Amersham) diluted in blocking buffer (1:20,000) for 1 hour at room temperature. After washes, blots were incubated for 5 minutes with a chemiluminescent substrate. For normalization purposes, blots were incubated in stripping buffer (Thermo Scientific), washed with TBS-T, blocked at room temperature for 2 hours, and incubated in anti-GAPDH (Thermo Scientific MA5-15738-HRP) diluted in blocking buffer (1:4000). Images presented represent reproducible conclusions from two completely independent experiments.

### Microscopy

A Zeiss 510 META Confocal Laser Scanning microscope equipped with Carl Zeiss Zen2009 image software was used to view and image immunofluorescence slides. For quantification of immunofluorescence intensity, four independent images from two independent experiments were obtained for each condition (Negative miR, miR-31, miR-200a, Mix).

### Immunohistochemistry of human retinoblastomas

Four human retinoblastomas, formalin-fixed paraffin-embedded (FFPE) products of enucleation were obtained from Ann & Robert H. Lurie Children's Hospital of Chicago and Northwestern University Feinberg School of Medicine. In addition, four unstained human retinoblastoma tissue arrays involving up to 26 retinoblastoma cores (from 13 individuals) and one hematoxylin and eosin stained retinoblastoma array (from the same block) were purchased from US Biomax (Catalog# BC35111a). Immunohistochemistry of was performed by an automated system at the Pathology Core Facility of the Robert H. Lurie Comprehensive Cancer Center of Northwestern University. The Leica Bond Polymer Refine Detection (Catalog # DS9800) and the Leica Bond™ Ready-To-Use Negative Control, (negative rabbit, Catalog# PA0777) were used for this technique. Primary antibodies (15 minute incubation) used for this procedure were DLL3 (Acris AP51274PU-N, 1:25), ACOT7 (Abcam ab85151, 1:50), and SYK (Abcam ab40781, 1:200); secondary incubation (8 minutes).

## Results

### Expression of miRNAs-31 and -200a is downregulated in retinoblastoma

**Fig 1:**
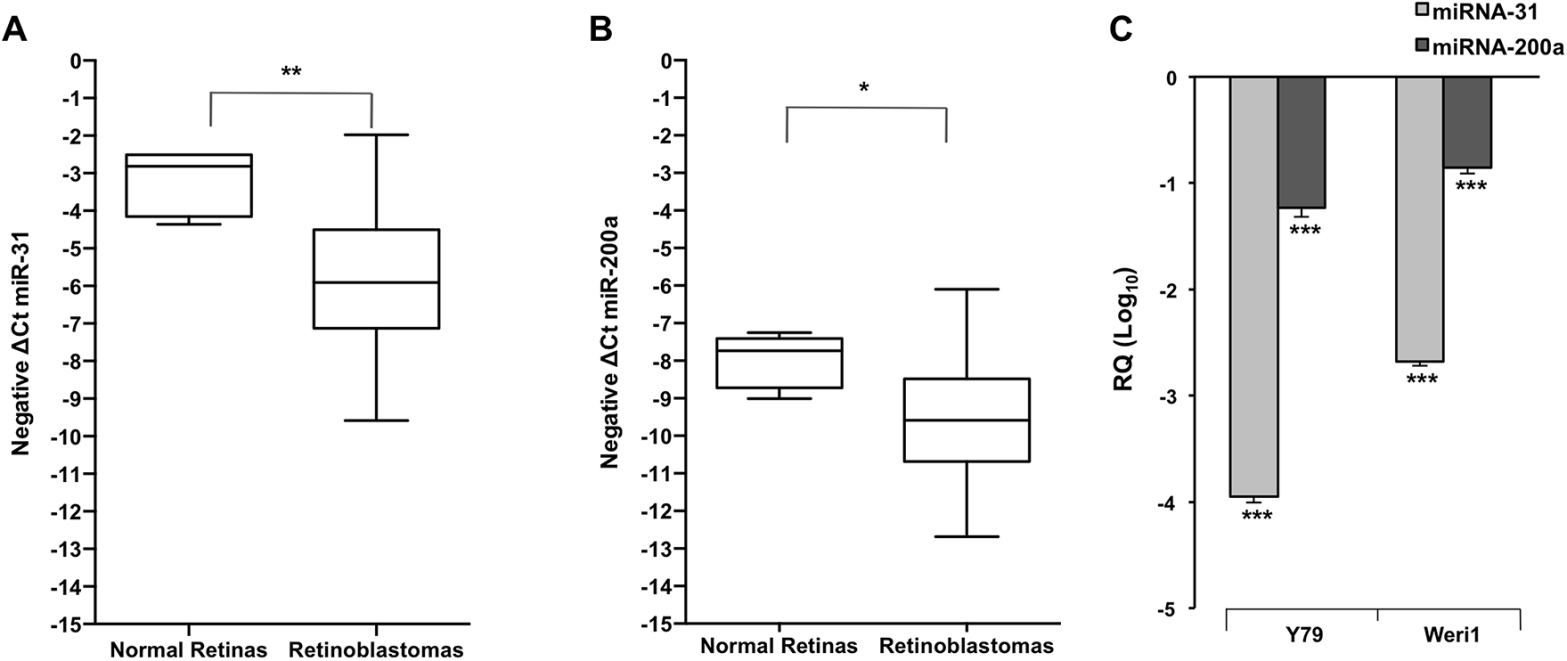
MicroRNAs- 31 and -200a are significantly downregulated in human retinoblastoma tumors and cell lines. (A) The expression level of miR-31 was measured in retinoblastomas as compared to normal pediatric retinas. (B) The expression level of miR-200a was measured in human retinoblastomas as compared to normal pediatric retinas. (C) The expression level of miR-31 and miR-200a was measured in human retinoblastoma cell lines (Y79, Weri1) as compared to normal pediatric retinas. Data represents mean and standard deviation from two independent experiments in triplicate. *denotes p< 0.05, ** denotes p< 0.003, *** denotes p<0.0005.

For validation of miRNA-31 and miRNA-200a expression in human retinoblastomas, we analyzed a cohort of 21 primary human retinoblastomas. Our cohort was demographically consistent with the literature, with patients presenting at a median age of 21 months. From this tumor cohort (where information was available), the percentage of patients who presented with evidence of anterior chamber invasion, choroid invasion, and optic nerve invasion was 10%, 38.1%, and 61.9%, respectively, (S1A Fig). Among the group of patients with evidence of optic nerve invasion (13/21), five individuals presented with optic nerve invasion alone, while eight individuals presented with additional invasion of the choroid. The majority of retinoblastomas within our cohort were also poorly differentiated (13/20 patients), (S1B Fig). We performed real-time PCR and confirmed that miRNA-31 and miRNA-200a are significantly reduced in twenty-one human retinoblastomas as compared to five normal pediatric retinas (Fig 1A and 1B). Next, we evaluated the expression of miRNA-31 and miRNA-200a in two human retinoblastoma cell lines, Y79 and Weri1 (Fig 1C). In comparison to three normal pediatric retinas, both miRNA-31 and miRNA-200a were significantly downregulated in both cell lines.

### miRNAs-31 and -200a target similar signaling pathways in retinoblastoma

To improve our understanding of the potential functional consequences of loss of miRNAs-31 and-200a expression, we employed GOmir [23] and MetaCore^TM^ (Thomas Reuters) to identify miRNA-mRNA interactions of particular interest for retinoblastoma studies (S1 Table). Using GOmir, we obtained 305 putative miRNA-31 mRNA targets and 656 putative miRNA-200a mRNA targets (S2 Table). In order to identify significant pathways enriched by these targets, we input this data into MetaCore^TM^ (Thomas Reuters). MicroRNA-target gene-prediction analyses indicate that miRNA-31 and miRNA-200a could significantly target genes that are expressed in primary retinoblastomas, such as TIAM1 [29] (S2 Table). Although GOmir only reports T-cell lymphoma invasion and metastasis 1 (*TIAM1*) as a miRNA-200a target, it was previously validated as a miRNA-31 target in colon cancer cells [30]. Our analysis *in silico* further suggests that both miRNAs (-31 and -200a) target other cancer-associated genes such as protein kinase C epsilon (*PRKCE*) [31]. Predicted targets of miRNA-31 suggests it has a role in cell trafficking, as it is most significantly enriched in pathways important for clathrin-coated vesicle formation, in addition to cytoskeleton remodeling (S1 Table). We further observed that putative targets of miR-200a are most significantly enriched in developmental signaling, apoptosis, survival, cell cycle, and gene translation (S1 Table). Notably, targets of both miRNAs (-31, -200a) appear to be significantly involved in cell-adhesion-Ephrin signaling (S1 Table). Eph receptors/ephrin proteins significantly influence proper axonal guidance required for migrating cells [32]. Altogether, our real-time PCR studies coupled with our analyses *in silico* supports our hypothesis that miRNA-31 and miRNA-200a have an important role in retinoblastoma proliferation.

### Overexpression of miRNAs-31 and -200a reduces proliferation of Y79 retinoblastoma cells

**Fig 2:**
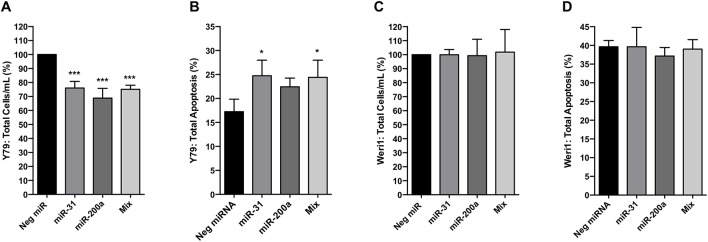
Increased expression of miRNAs-31 and -200a significantly reduces Y79 cell proliferation, but not Weri1 cell proliferation. Bar demonstrates percentage in total cells per mL for Y79 (A) and Weri1 (C) at 96 hours post-transfection with indicated miRNA mimic(s) as compared to controls (negative miR mimic). Total percent apoptosis (sum of early and late apoptotic percentage of cells) was determined in Y79 (B) and Weri1 (D) cells. Data represents mean and standard deviation from three independent experiments with triplicate samples. * denotes p<0.05, *** denotes p < 0.001.

We next evaluated the extent to which overexpression of miRNA-31, miRNA-200a, or concurrent overexpression of both miRNAs affects retinoblastoma cell proliferation *in vitro*. To do this, we used miRNA mimics to transiently increase expression of miRNAs-31 and/or -200a in Y79 and Weri1 retinoblastoma cells (S2 Fig). We demonstrate that overexpression of miRNA-31, miRNA-200a, or both miRNAs together (Mix) significantly reduce Y79 retinoblastoma proliferation as compared to control-treated cells by 23.94%, 31.13%, and 24.84%, respectively, at 96 hours post-transfection (Fig 2A). However, this effect was not observed in Weri1 cells treated similarly (Fig 2C). We next examined if there was an increase of apoptosis, including both early and late stage apoptotic cells, under the same conditions. Using annexin V and 7-AAD as markers, we detected a significant increase in levels of total cell apoptosis in Y79 cells after miR-31 or miR-200a overexpression (Fig 2B). This result was also observed in cells co-expressing both miRNAs-31 and -200a (Fig 2B). Weri1 cells do not demonstrate increased levels of total apoptosis when treated similarly (Fig 2D). Conversely, we expressed miRNA inhibitors to evaluate if exerting a greater loss of miR-31 and miR-200a expression had any consequence in their ability to proliferate (S3 Fig). Data shows no resultant effect in Y79 or Weri1 cells, suggesting the impact stemming from loss of the two miRNAs had reached a threshold.

### miRNAs-31 and -200a overexpression negatively regulate genes associated with cell proliferation and survival

In order to elucidate the genetic mechanisms underlying our observations, we used a gene expression array (>47,000 probes) to identify those genes most differentially expressed after increasing miRNA-31 or miRNA-200a expression individually, or when co-overexpressed together (Mix) in Y79 cells, as compared to controls. From our bioinformatics analysis of these data, we observed that the resultant gene expression profiles (Table 1 and S1 Data) included predicted targets (e.g. *STK40*, *CCND2*, *ACOT7*, *PPP6C;* S2 Table) and non-predicted target genes (e.g. *CALM1*, *DLL3*, *PDHA1*, *PIGY)* of miRNAs-31 and/or -200a.

**Table 1:** Top 10 genes downregulated after increased microRNA-31 and -200a expression in Y79 retinoblastoma cells.

microRNA	Downregulated mRNAs		
	Gene Symbol	Fold Change	p-Value
miRNA-31	STK40	-2.83	9.12E-16
	CALM1	-2.52	8.82E-17
	GNGT1	-2.46	3.79E-12
	ARHGEF2	-2.30	1.16E-13
	FAM127A	-2.22	6.24E-14
	NSF	-2.14	3.85E-18
	KCNF1	-2.06	2.15E-14
	ID3	-1.97	4.54E-08
	PPP6C	-1.97	1.12E-12
	DLL3	-1.95	1.11E-11
miRNA-200a	ACOT7	-2.58	1.72E-13
	SNRPB2	-2.25	5.62E-15
	NNAT	-2.15	2.00E-11
	CCND2	-2.12	1.82E-08
	SRM	-2.12	8.07E-17
	PDHA1	-1.94	6.40E-10
	DCP2	-1.91	1.67E-13
	TNPO1	-1.91	1.95E-13
	DLL3	-1.91	1.94E-11
	KCTD20	-1.90	3.93E-14
Mix	NNAT	-2.43	1.40E-12
	STK40	-2.36	3.42E-14
	ARHGEF2	-2.33	8.42E-14
	CALM1	-2.31	6.07E-16
	DLL3	-2.28	2.34E-13
	SRM	-2.05	1.87E-16
	ST13	-2.00	1.04E-09
	KCTD20	-1.92	2.82E-14
	PPT1	-1.90	5.60E-12
	PIGY	-1.90	1.38E-11

Top 10 genes downregulated after increased microRNA-31 and -200a expression in Y79 retinoblastoma cells. Genes were identified as being downregulated on the basis of a statistically significant fold change (p-value < 0.05) in expression level for the comparisons between the indicated miRNA overexpressing samples and its control (negative miRNA).

Among the ten most significantly downregulated genes following miRNA-31 overexpression, we identified targets that could be important for retinoblastoma progression (Table 1 and S1 Data). These include serine/threonine kinase 40 (*STK40*), which encodes an inflammatory regulator (mediated by suppression of NF-κB), previously shown to be a direct target of miRNA-31 [33]. STK40 is also a regulator of tumor protein p53 (*TP53*) transcription [34]. We further noted downregulation of protein phosphatase 6 catalytic subunit (*PPP6C*), whose encoding protein is significantly correlated with malignant mesothelioma proliferation and also previously validated as a direct target of miRNA-31 [35]. We also observed downregulation of *DLL3* following overexpression of miRNA-31 or miRNA-200a. *DLL3* mRNAs encode delta-like 3, an N-myc transcriptional target linked to abnormal hyper-proliferation in the brain [36]. The ability of two distinct miRNAs that can functionally repress *DLL3* may indicate that regulation of *DLL3* is of high significance. Continuing our screen for targets that may have triggered Y79 sensitivity to miRNA overexpression (Fig 2), we also identified repression of acyl-CoA thioesterase 7 (*ACOT7*) following miR-200a overexpression. This was particularly notable as ACOT7 is highly important for protecting a cell against internal toxic conditions [37]. Therefore, it is conceivable that downregulation of *STK40*, *PPP6C*, *DLL3*, *or ACOT7* may have prompted an inhibitory effect on proliferation we observed in Y79 cells (Fig 2A).

**Fig 3:**
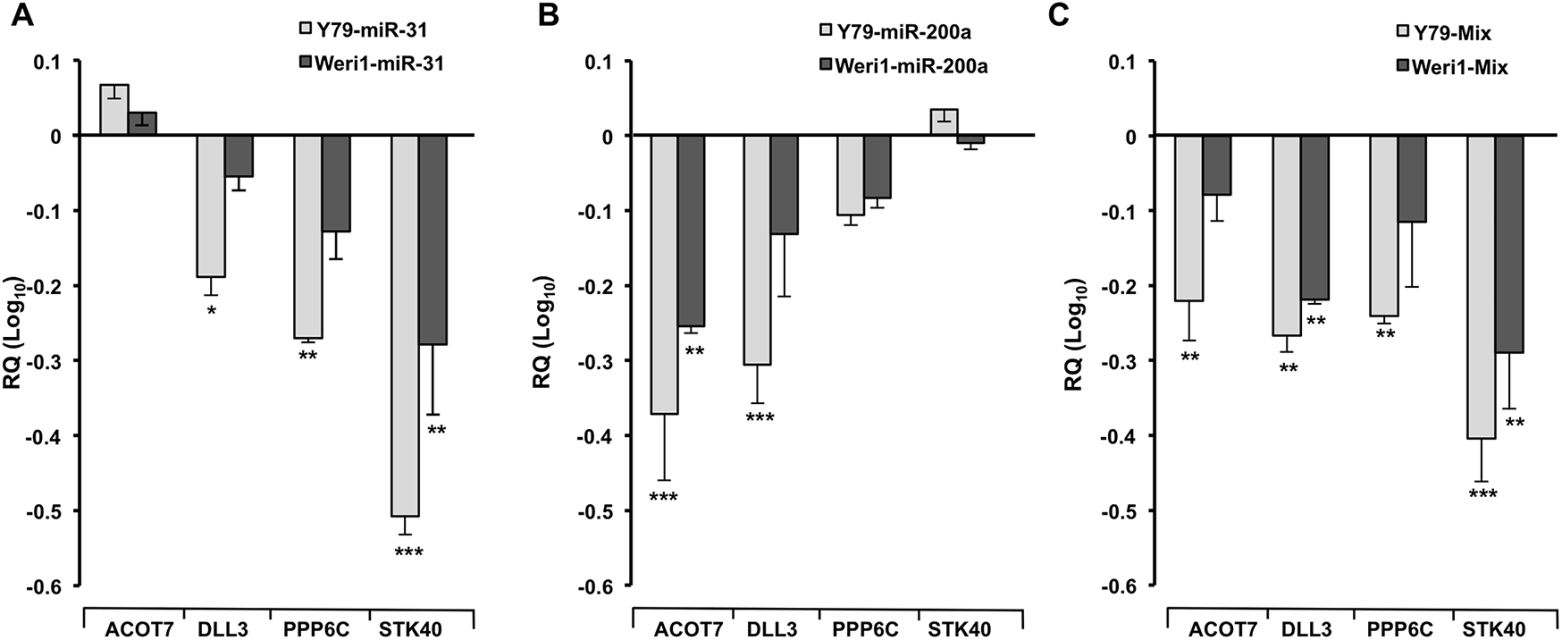
Increased expression of miRNAs-31 and -200a negatively regulates genes associated with proliferation and survival. (A- C) Expression of *ACOT7*, *DLL3*, *PPP6C*, and *STK40* mRNAs as measured by TaqMan qRT-PCR in human retinoblastoma cells (Y79 and Weri1) after transient overexpression of miR-31 (A), miR-200a (B), and co-transfected miRs-31 and -200a, “Mix” (C) as compared to negative control miRNA overexpressing cells. Data represents mean and standard deviation from two independent experiments. * denotes p< 0.05, ** denotes p< 0.01, *** denotes p< 0.0005.

Validation of *STK40*, *PPP6C*, and *DLL3* knockdown by miRNA-31, miRNA-200a, or miRNA-31/-200a (Mix) was confirmed using real-time PCR in Y79 cells (Fig 3). To improve our understanding of miRNA-31 and/or -200a mediated regulation in retinoblastoma cells, we also evaluated the extent of repression of *STK40*, *PPP6C*, and *DLL3* in Weri1 cells to determine if these miRNA-mRNA interactions remained intact. We did not observe a statistically significant knockdown of *DLL3* in Weri1 cells, in contrast to Y79-miR-31 or Y79-miR-200a-expressing cells (Fig 3A and 3B), suggesting a miRNA-target interaction unique to Y79 cells. Among the four candidate mRNAs examined, *STK40* was the single mRNA significantly reduced in Weri1 cells following miR-31 overexpression. As *STK40* is not a predicted target of miR-200a using GOmir [23] (S2 Table), nor was its expression found to be different in our profiling arrays, we did not observe knockdown of *STK40* in cells overexpressing miRNA-200a, as we expected (Fig 3B).

**Fig 4:**
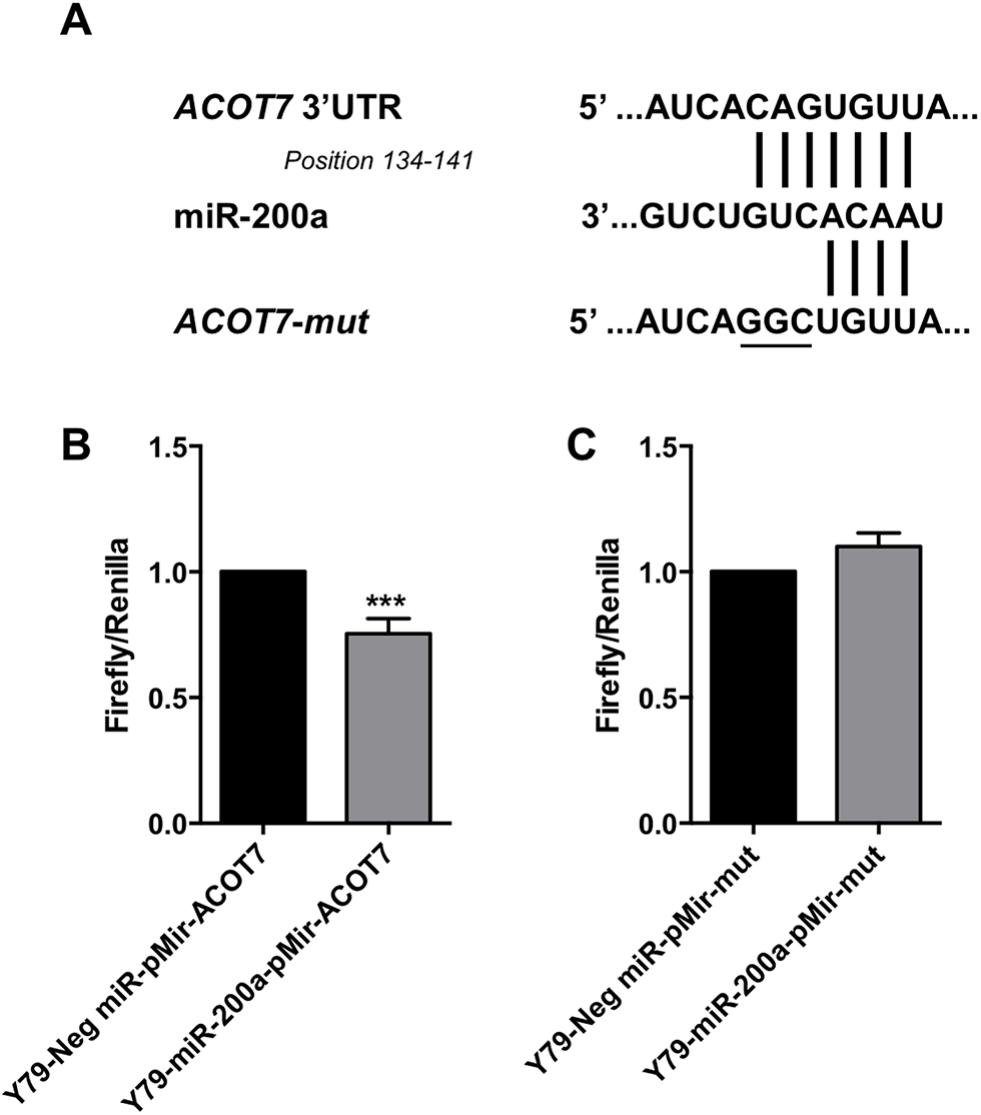
*ACOT7* is a direct target of miRNA-200a. (A) The nucleotide sequence of the putative miR-200a binding site within the 3’UTR of *ACOT7*. As a negative control, a mutated version of the binding site (‘ACOT7-*mut*’) contains three different nucleotides (underlined). (B) Y79 retinoblastoma cells, transfected with a miR-200a mimic or a negative (control miR) mimic, were co-transfected with plasmid vectors containing a firefly luciferase gene coding region upstream of either the ACOT7-3’UTR (pMir-ACOT7) or the mutated ACOT7- 3’UTR (pMir-mut), in addition to a renilla luciferase plasmid for normalization purposes. Data represents mean and standard deviation from independent triplicates from two experiments. *** denotes p< 0.0001.

The capacity for miR-200a to repress *ACOT7* in retinoblastoma cells suggests a novel contributory role of ACOTs in retinoblastoma progression. Therefore, we further validated knockdown of *ACOT7* after miRNA-200a overexpression in Y79, which we observed to also occur Weri1 cells (Fig 3B). MiRNA-31 did not significantly reduce expression levels of *ACOT7*, as it is not predicted to be among its targets (Fig 3A and S2 Table). To further validate this miRNA-mRNA binding interaction, we utilized a firefly luciferase reporter vector with the ACOT7 3’ untranslated region (UTR) (pMir-ACOT7, Fig 4A). This vector was co-transfected into Y79 cells receiving a negative miR mimic (control) or a miR-200a mimic (Fig 4B). As compared to controls, we observed a significant decrease in luciferase activity in miR-200a overexpressing cells. Furthermore, we cloned a mutated version of the ACOT7 3’ untranslated region (UTR) into a second luciferase reporter (pMir-mut, Fig 4A and 4C). As compared to controls, Y79 cells overexpressing miR-200a exhibited no significant difference of pMir-mut luciferase activity. This data confirms that a specific binding site sequence interaction is required for miR-200a mediated regulation of *ACOT7*.

Co-overexpression of miRNAs-31 and -200a (mix) in Y79 cells was sufficient to sustain a significant knockdown of each mRNA evaluated (*ACOT7*, *DLL3*, *PPP6C*, *STK40*; Fig 3C). We also observed similar significant repression of *STK40* in Weri1 cells.

**Fig 5:**
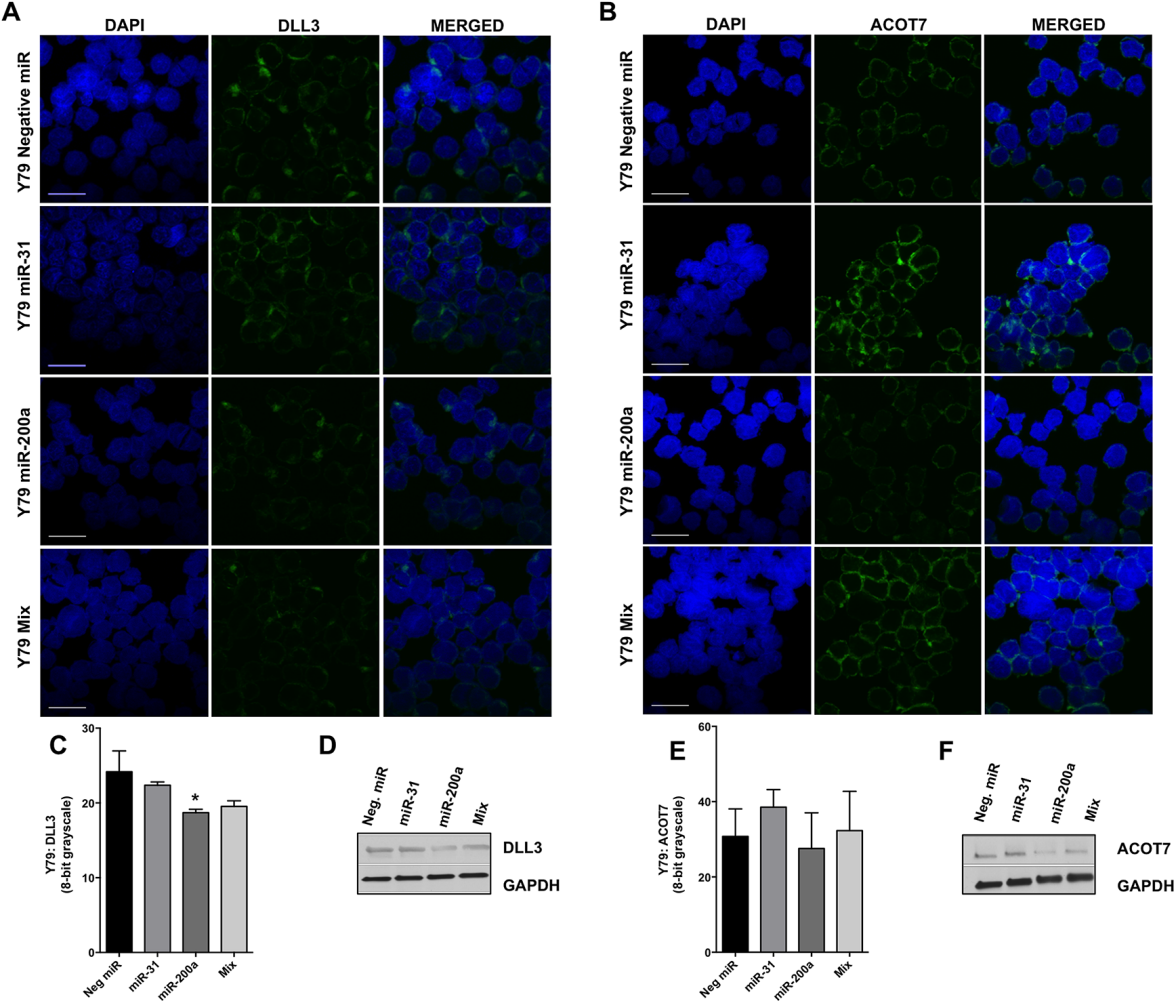
DLL3 and ACOT7 expression in Y79 cells following overexpression of miRNAs-31, -200a, or when overexpressed together (Mix). Immunofluorescence staining of DLL3 (A) and ACOT7 (B) in Y79 cells transfected with a negative miRNA (control), miRNA-31, miRNA-200a, and miR-31/-200a (Mix). Quantification of immunofluorescence intensity of DLL3 (C) and ACOT7 (E); bar represents mean and standard deviation from two independent experiments with quadruplicate measurements. Western blot analysis of DLL3 (D) and ACOT7 (F) in Y79 cells transfected with a negative miRNA (control), miRNA-31, miRNA-200a, and miR-31/-200a (Mix). * denotes p< 0.05. Scale bar 20 μm.

To support our gene expression studies, we used immunofluorescence to determine the extent to which overexpression of miRNAs-31 and/or -200a reduces endogenous protein levels of their respective mRNA targets in Y79 cells (Fig 5). We observed that miRNA-200a overexpression leads to reduction of DLL3 in Y79 cells (Fig 5A). Using 8-bit grayscale as a measure of signal intensity, we found this feature to be statistically significant (Fig 5C). A substantial decrease was not observed using western blotting (Fig 5D). In contrast to our real-time PCR data that demonstrates miRNA-31 can significantly repress *STK40* or *PPP6C* in Y79 cells, we did not observe any difference in STK40 or PPP6C protein expression as compared to controls, which suggests regulatory changes in the abundance of these mRNAs (mediated by miR-31) are inconsequential (S4 Fig). Although we did not detect a significant difference of ACOT7 in Y79 cells using immunofluorescence (Fig 5B and 5E), we did observe a substantial decrease of ACOT7 following miRNA-200a overexpression using western blotting (Fig 5F), which was sustained under the Y79-Mix condition.

**Fig 6:**
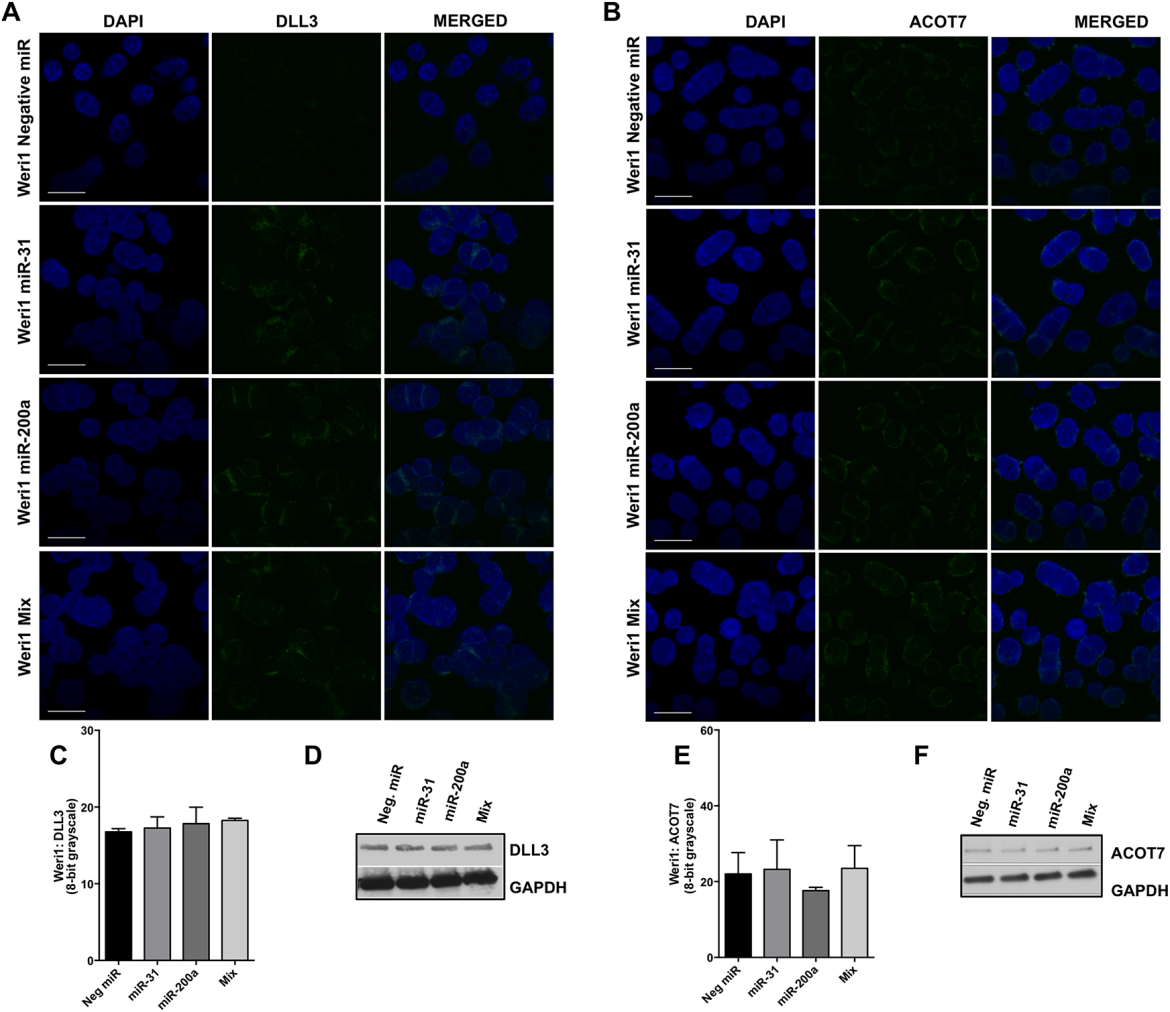
DLL3 and ACOT7 expression in Weri1 cells following overexpression of miRNAs-31, -200a, or when overexpressed together (Mix). Immunofluorescence staining of DLL3 (A) and ACOT7 (B) in Weri1 cells transfected with a negative miRNA (control), miRNA-31, miRNA-200a, and miR-31/-200a (Mix). Quantification of immunofluorescence of DLL3 (C) and ACOT7 (E); bar represents mean and standard deviation from two independent experiments with quadruplicate measurements. Western blots of DLL3 (D) and ACOT7 (F) of Weri1 cells transfected with a negative miRNA (control), miRNA-31, miRNA-200a, and miR-31/-200a (Mix). Scale bar 20 μm.

Similarly, we further evaluated these interactions in Weri1 cells (Fig 6 and S5 Fig). We observed no reduction of DLL3, ACOT7, PPP6C, and STK40 protein levels following miRNA-31 and/or -200a overexpression.

### Selective targeting of *DLL3* or *ACOT7* recapitulates miR-31 and/or miR-200a-mediated suppression of Y79 cell proliferation

**Fig 7:**
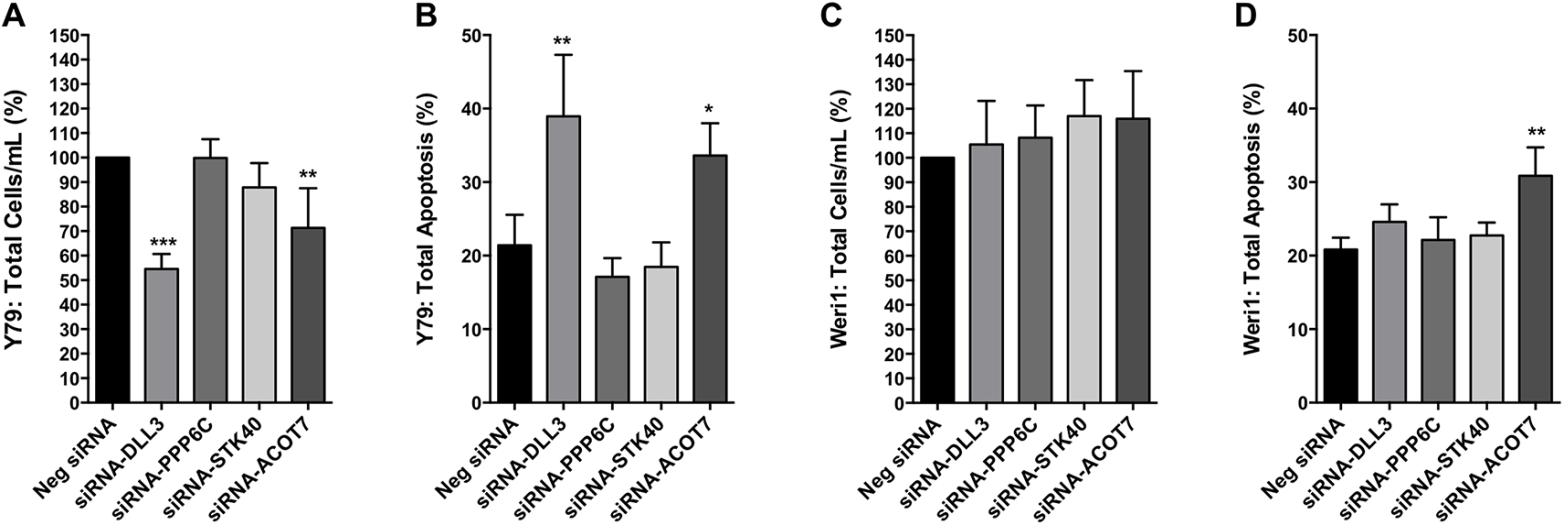
Reduced *DLL3* or *ACOT7* inhibits Y79 cell proliferation. Bar demonstrates percentage in total cells per mL for Y79 (A) and Weri1 (C) at 120 hours post-transfection with indicated siRNAs for *DLL3*, *PPP6C*, *STK40*, and *ACOT7*. Data represents mean and standard deviation from four independent experiments with triplicate samples. Total percent apoptosis (sum of early and late apoptotic percentage of cells) was determined in Y79 (B) and Weri1 (D) cells. Data represents mean and standard deviation from three independent experiments with triplicate samples. * denotes p< 0.05, ** denotes p< 0.005, *** denotes p < 0.0001.

To determine if selective reduction of endogenous *DLL3*, *PPP6C*, *STK40*, or *ACOT7* could recapitulate the inhibitory effect on proliferation mediated by miRNAs-31 and/or -200a, we transiently introduced small interfering RNAs (siRNAs) that are designed to target specific mRNAs for degradation (Fig 7). We observed that transfection of a siRNA specific for *DLL3* in Y79 cells led to a significant reduction in total cell proliferation (-45.4%) as compared to cells expressing a non-specific siRNA (Fig 7A). This result was not observed in Weri1 cells (Fig 7C). We also observed a significant reduction (-28.63%) in total cell proliferation following direct targeting of *ACOT7* mRNAs in Y79 cells (Fig 7A), but observed no statistically significant effect in Weri1 cells (Fig 7C). We did not detect significant changes in cell proliferation following reduction of *PPP6C* or *STK40* in either retinoblastoma cell line (Fig 7A and 7C). We further detected a significant (+17.51%) increase in Y79 cell apoptosis following siRNA-DLL3 transfection, as compared to controls (Fig 7B). However, transfection of *DLL3* siRNA had no significant impact on Weri1 cell apoptosis (Fig 7D). Transfection of a siRNA specific to *ACOT7* also conferred a similar phenotypic result in Y79 and Weri1 cells, as we observed an increase of total cell apoptosis by approximately 12.21% (Fig 7B) in Y79 cells, and an increase of 10.12% in Weri1 cells (Fig 7D).

Next, we used a second set of siRNAs (siRNAs#2) against *DLL3* or *ACOT7* mRNAs to determine if targeting alternative exon(s) would confer similar results (S6 Fig). In contrast to the first siRNA targeting *DLL3*, we observed a significant increase (+17%) in Weri1 cell proliferation (S6C Fig), which could suggest that expression of *DLL3* and proliferation are inversely correlated in a phenotypically less aggressive retinoblastoma cell line. Following transfection with an alternative siRNA against *ACOT7* in Y79 cells, we report a greater significant reduction (-39.16%) in proliferation than observed previously, in addition to a moderate increase in total apoptosis, but not statistically significant (S6A and S6B Fig). We attributed the differential phenotypic differences exhibited by these alternative siRNAs to their knockdown efficiency, as suggested by qRT-PCR (S7 Fig).

**Fig 8:**
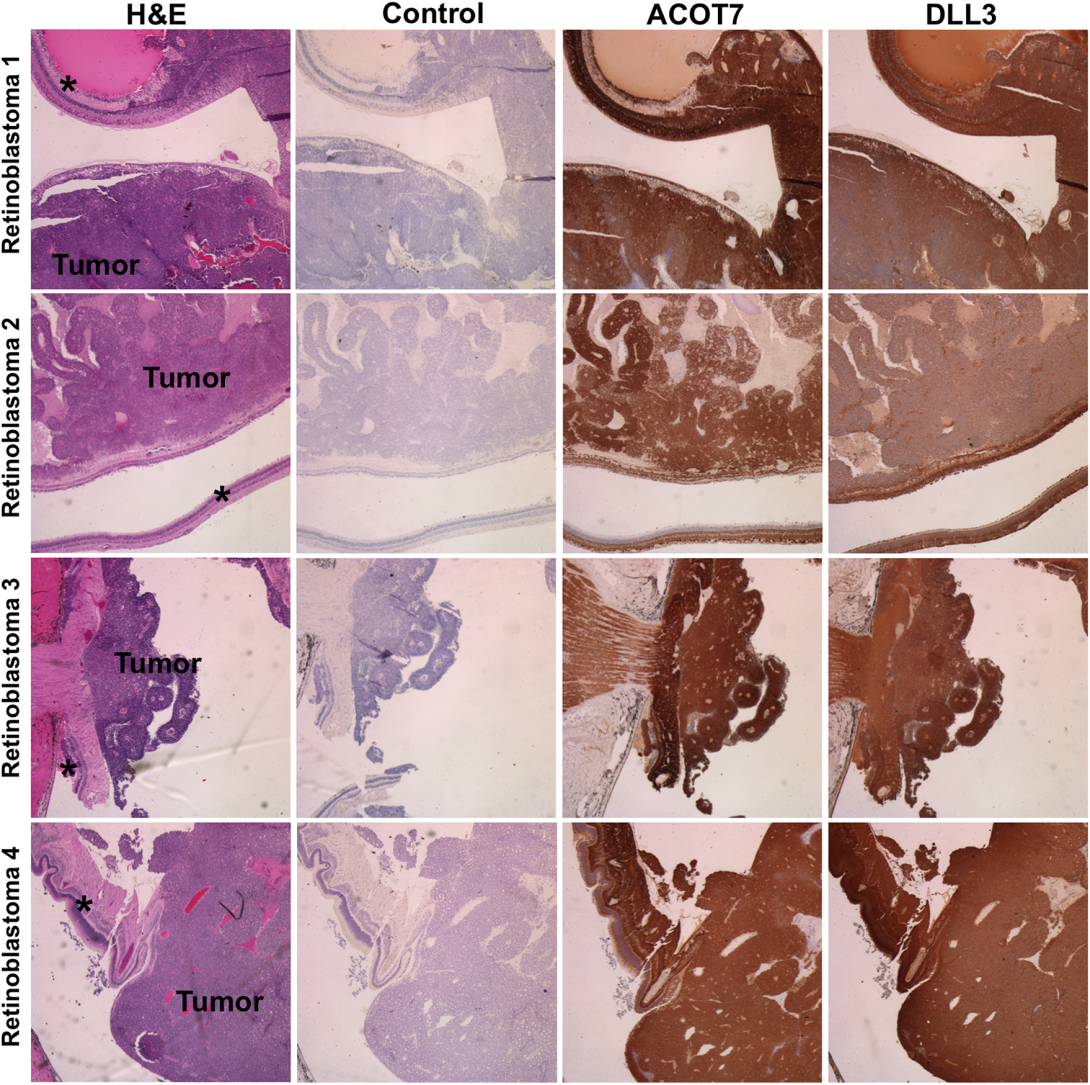
Human retinoblastomas express ACOT7 and DLL3. Immunohistochemistry was performed on four retinoblastoma samples obtained from four individual pediatric patients. Hematoxylin and eosin staining of each retinoblastoma (retinoblastoma 1–4) shows identified tumor (Tumor) and adjacent retina (denoted with an asterisk). Control staining served as a negative control (no primary; secondary antibody only; hematoxylin counterstain). In four of four cases, retinoblastomas display expression of ACOT7 and DLL3.

To establish clinical relevance of ACOT7 and DLL3, we performed immunohistochemistry on four human pediatric retinoblastomas representing individual patients (Fig 8). As a positive control for this study, we stained for the proto-oncogene spleen tyrosine kinase, SYK (data not shown), previously identified to be expressed in primary retinoblastomas [8]. From within this cohort, we observed that ACOT7 and DLL3 are positively expressed in each retinoblastoma tumor evaluated, in addition to adjacent retina. We confirmed this finding in a human retinoblastoma tissue array (13 individual patients with duplicate cores, S9 Fig). Expression of ACOT7 and DLL3 was observed in a majority of retinoblastomas available for analysis (S9A and S9B Fig), and were also expressed in retinal tissues (n = 5), (S9C Fig).

## Discussion

Based upon previous miRNA profiling reports [12, 16], we hypothesized that downregulation of miRNA-31 and miRNA-200a may be an important contributing factor for retinoblastoma proliferation. Using real-time PCR, we confirmed that human retinoblastomas exhibit loss of miRNA-31 and -200a expression as compared to normal pediatric retinas (Fig 1). Our early *in silico* analyses indicated that miRNA-31 and -200a are each capable of regulating pathways important for development, proliferation, apoptosis, cell cycle, and cell adhesion (S1 Table). By overexpressing miRNA-31 and -200a *in vitro*, we demonstrated that miRNA-31 and/or miRNA-200a significantly reduce Y79 cell proliferation, but not Weri1 cell proliferation (Fig 2). Furthermore, our work is the first to demonstrate that overexpression of miRNA-31 and/or miR-200a results in differential gene expression patterns of *ACOT7*, *DLL3*, *PPP6C*, and *STK40* between two phenotypically different retinoblastoma cell lines (Fig 3).

Among the most downregulated mRNAs in Y79 cells, *ACOT7* and *DLL3* represent novel therapeutic targets for future retinoblastoma studies, which we found to be expressed in primary retinoblastomas and retinas (Fig 8 and S9 Fig), and overexpressed in retinoblastoma cell lines as compared to normal retinas (S8 Fig). Acyl-CoA thioesterases (ACOTs) belong to a class of enzymes responsible for cytoplasmic long-chain acyl-CoA hydrolysis in mammals [37]. In particular, Ellis et al., have found ACOT7 to be expressed in multiple tissues that include the murine eye. They also reported that high, yet, selective expression of ACOT7 in the brain is required for preventing toxic accumulation of cellular free fatty acids, phospholipids, and triacylglycerols. Our second target, *DLL3*, is an N-myc transcriptional target [36], and is of particular interest because Y79 cells display amplification of the proto-oncogene, *MYCN* [38]. Moreover, *MYCN* amplification, a rare feature of most primary human retinoblastomas, occurs with greater incidence in retinoblastomas without *RB1* mutations [39]. Zhao and colleagues found that *DLL3* is negatively regulated by Huwe1, an ubiquitin ligase that is critical for inhibiting aberrant neuronal proliferation and differentiation in the developing brain [36]. We report these findings regarding *ACOT7* and *DLL3* to be consistent in Y79 cells, but not in Weri1 (Fig 7). The inability of miRNAs-31 and -200a to reduce Weri1 cell proliferation could be attributed to insufficient repression of a repertoire of genetic factors that might include *DLL3* and/or *ACOT7* (Fig 3).

The differential results between Y79 and Weri1 cells could be attributed to their differing genetic background, as Y79 cells were derived from a child with familial retinoblastoma [21], unlike Weri1 cells which were derived from a child with no family history of retinoblastoma [22]. These cell lines also differ in their ability to invade ocular structures [40, 41] and in their cell growth properties [22, 42]. Although Weri1 cells can locally invade structures such as the lens and choroid, these cells do not invade the optic nerve [40, 41]. Jo and colleagues have previously reported differential miRNA profiles between retinoblastoma cell lines (Y79, SNUOT-Rb1) with different proliferation and adherence patterns [43]. Retinoblastomas that are regulated by distinct miRNAs, such as miRNA-31 and miRNA- 200a, may enable the development of phenotypic differences in their ability to proliferate and invade local and distant structures, such as the differences that exist between Weri1 and Y79 cells. Both cell lines (Y79, Weri1) exhibit inactivation of *RB1* [6, 44] and they do not express hypophosphorylated or phosphorylated RB1 protein [39]. However, there exist recurrent gene expression variations, such as the levels of *MYCN*, where Weri1 cells display a 2.2 fold increase, while Y79 cells exhibit much greater amplification (53 fold) as compared to normal retinas [39]. Such distinctions could contribute to phenotypic differences.

In order to overcome obstacles to their long-term survival, we hypothesized that some retinoblastomas may rely upon significantly reducing miRNA-31 and miRNA-200a expression. In this study, we observed that increasing expression of miRs-31 and/or -200a reduced cell proliferation of Y79, but not Weri1 cells. From these observations, it is plausible that as retinoblastoma progresses, overexpression of *DLL3* and *ACOT7* could be characteristic features. In future studies, it will be important to identify the underlying factors responsible for this differential result, especially in light of well-documented phenotypic differences between the cell lines, that includes differential cell growth rates [22, 42] and invasive capabilities [40, 41]. This work has the potential to contribute a greater insight towards the development of more specific treatments for patients with aggressive retinoblastomas.

## Supporting Information

S1 Data Most significantly downregulated genes after increased microRNA-31 and -200a expression in Y79 retinoblastoma cells. Genes were identified as being downregulated on the basis of a statistically significant fold change (p-value < 0.05) in expression level for the comparisons between the indicated miRNA overexpressing samples and its control (negative miRNA).
(XLSX)

S1 Fig Important clinical features of patients with retinoblastoma. (A) Bar demonstrates percentage of individuals who presented with evidence of anterior chamber invasion (2/20), choroid invasion (8/21), and optic nerve invasion (13/21 patients), in addition to those who presented with no evidence of invasion (8/21). (B) Bar demonstrates the percentage of individuals who presented with poor (13/20), mixed (2/20), moderate (2/20), or well-differentiated retinoblastomas (3/20).
(TIF)

S2 Fig Real-time PCR of miRNA-31 and/or -200a expression following miRNA mimic transfection in Y79 and Weri1 cells. Expression of miRNAs-31 (A,C) and -200a (B,D) as measured by TaqMan qRT-PCR in human retinoblastoma cells (Y79 and Weri1) after transient overexpression of miR-31, miR-200a, or co-transfected miRNAs-31 and -200a (Mix), as compared to negative control miRNA overexpressing cells. Data represents mean and standard deviation from two independent experiments. *** denotes p< 0.001.
(TIF)

S3 Fig Use of miRNA inhibitors in retinoblastoma cells does not impact proliferation. Bar demonstrates percentage in total cells per mL for Y79 (A) and Weri1 (C) at 96 hours post-transfection with indicated miRNA inhibitors for miRNA-31 and/or -200a. Total percent apoptosis (sum of early and late apoptotic percentage of cells) was determined in Y79 (B) and Weri1 (D) cells. Data represents mean and standard deviation from three independent experiments with triplicate samples.
(TIF)

S4 Fig PPP6C and STK40 expression in Y79 cells overexpressing miRNAs-31, -200a, or when overexpressed together (Mix). Immunofluorescence staining of PPP6C (A) and STK40 (B) in Y79 cells transfected with a negative miRNA (control), miRNA-31, miRNA-200a, and miR-31/-200a (Mix). Quantification of immunofluorescence of PPP6C (C) and STK40 (E); bar represents mean and standard deviation from two independent experiments with quadruplicate measurements. Western blot analysis from one experiment of PPP6C (D) and STK40 (F) in Y79 cells transfected with a negative miRNA (control), miRNA-31, miRNA-200a, and miR-31/-200a (Mix). Scale bar 20 μm.
(TIF)

S5 Fig PPP6C and STK40 expression in Weri1 cells overexpressing miRNAs-31, -200a, or when overexpressed together (Mix). Immunofluorescence staining of PPP6C (A) and STK40 (B) in Weri1 cells transfected with a negative miRNA (control), miRNA-31, miRNA-200a, and miR-31/-200a (Mix). Quantification of immunofluorescence of PPP6C (C) and STK40 (E); bar represents mean and standard deviation from two independent experiments with quadruplicate measurements.Western blot analysis from one experiment of PPP6C (D) and STK40 (F) in Y79 cells transfected with a negative miRNA (control), miRNA-31, miRNA-200a, and miR-31/-200a (Mix). Scale bar 20 μm.
(TIF)

S6 Fig Reduced expression of an alternative siRNA against *ACOT7* inhibits Y79 cell proliferation. Bar demonstrates percentage difference in total cells per mL for Y79 (A) and Weri1 (C) at 120 hours post-transfection with indicated alternative siRNAs (siRNA#2). Total percent apoptosis (sum of early and late apoptotic percentage of cells) was determined in Y79 (B) and Weri1 (D) cells. Data represents mean and standard deviation from three independent experiments with triplicate samples. * denotes p< 0.05.
(TIF)

S7 Fig Quantitative real-time PCR expression of *DLL3* and *ACOT7* following siRNA transfection. Expression of *ACOT7* (A-B) and *DLL3* (C-D) as measured by TaqMan qRT-PCR in human retinoblastoma cells (Weri1 and Y79) after transient expression of two distinct siRNAs, as compared to negative control siRNA expressing cells. Data represents mean and standard deviation from two independent experiments. * denotes p<0.05, ** denotes p< 0.01, *** denotes p< 0.001.
(TIF)

S8 Fig Quantitative real-time PCR expression of *DLL3* and *ACOT7* in retinoblastoma cell lines. Expression of *ACOT7* (A) and *DLL3* (B) as measured by TaqMan qRT-PCR in human retinoblastoma cells (Y79 and Weri1) as compared to normal retinas from three individuals. Data represents mean and standard deviation from two experiments with triplicate samples. *** denotes p< 0.0005.
(TIF)

S9 Fig ACOT7 and DLL3 are expressed in a retinoblastoma tissue array. Immunohistochemistry was performed on four retinoblastoma tumor arrays (US Biomax, Catalog BC35111a) for ACOT7 and DLL3. Hematoxylin and eosin staining of retinoblastoma (A) and retinal tissues (C) shows identified tumor (Tumor) and retina, respectively. Control staining served as a negative control (no primary, secondary antibody only, hematoxylin counterstain). (A) Four representative cores from the retinoblastoma tissue array. (B) ACOT7 was detected in 16/26 tumor cores (61.54%) available for analysis. DLL3 was detected in 14/20 tumor cores (70.00%) available for analysis. (C) ACOT7 and DLL3 were detected in 5/5 retinal tissues available for analysis, but shown is one representative specimen.
(TIF)

S1 Table *In silico* analysis indicates microRNAs-31 and -200a each target pathways important for retinoblastoma progression. Table demonstrates the ten most statistically significant pathways enriched for miRNA-31 or -200a targets.
(TIF)

S2 Table Predicted targets of miRNA-31 and miRNA-200a identified by GOmir. (PDF)

## Abbreviations

miRNAs: microRNAs
TLDA: TaqMan low density array
Ct: cycle threshold
ΔCT: Delta Ct
RQ: relative quantitation
FFPE: formalin-fixed paraffin-embedded
