# Supplementary figures and images for: Novel miRNA-31 and miRNA-200a-Mediated Regulation of Retinoblastoma Proliferation
Source: PLoS One. 2015 Sep 17;10(9):e0138366. doi: 10.1371/journal.pone.0138366 (PMC4574557; doi:10.1371/journal.pone.0138366)

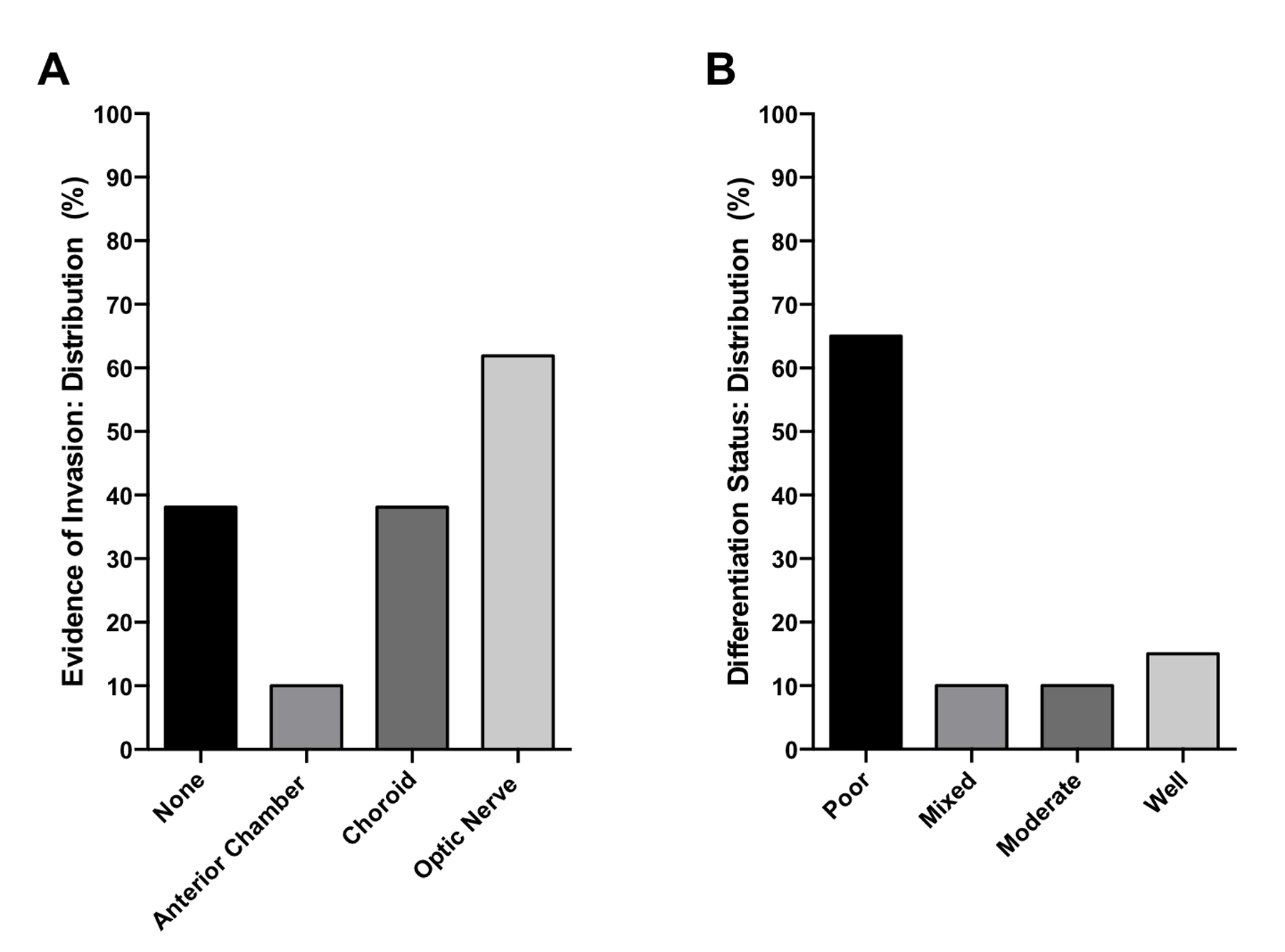

Supplement: S1 Fig — (A) Bar demonstrates percentage of individuals who presented with evidence of anterior chamber invasion (2/20), choroid invasion (8/21), and optic nerve invasion (13/21 patients), in addition to those who presented with no evidence of invasion (8/21). (B) Bar demonstrates the percentage of individuals who presented with poor (13/20), mixed (2/20), moderate (2/20), or well-differentiated retinoblastomas (3/20). (TIF) [file pone.0138366.s002.tif]

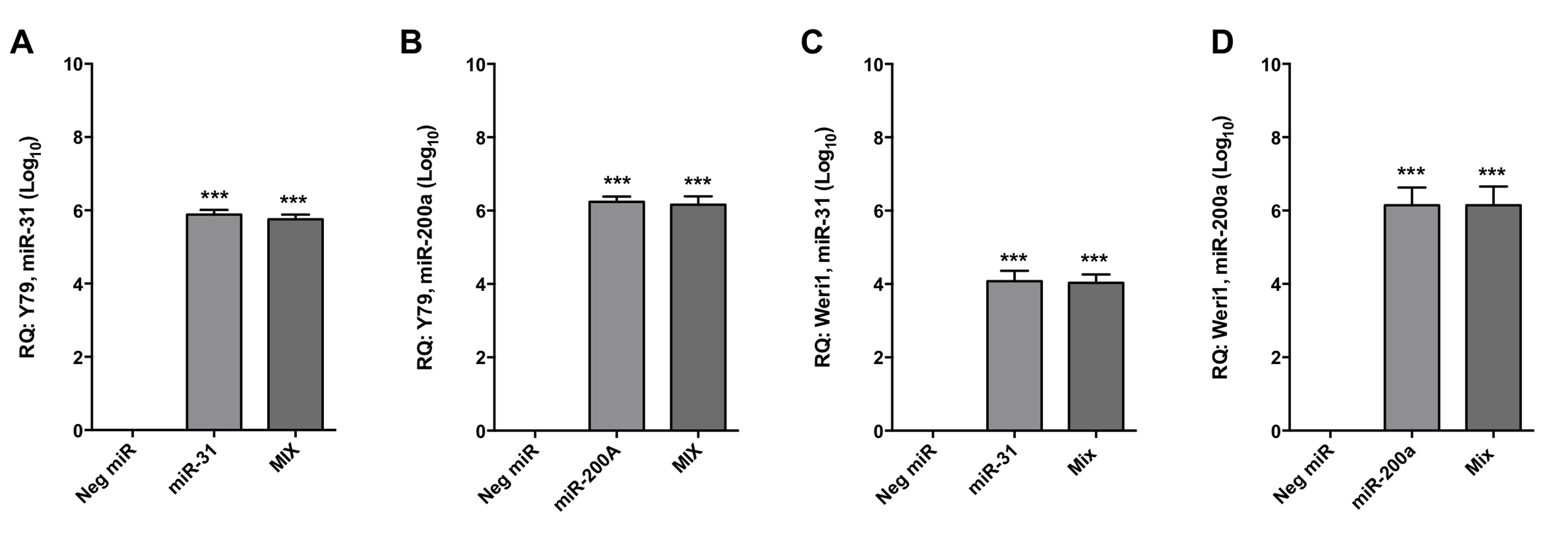

Supplement: S2 Fig — Expression of miRNAs-31 (A,C) and -200a (B,D) as measured by TaqMan qRT-PCR in human retinoblastoma cells (Y79 and Weri1) after transient overexpression of miR-31, miR-200a, or co-transfected miRNAs-31 and -200a (Mix), as compared to negative control miRNA overexpressing cells. Data represents mean and standard deviation from two independent experiments. *** denotes p< 0.001. (TIF) [file pone.0138366.s003.tif]

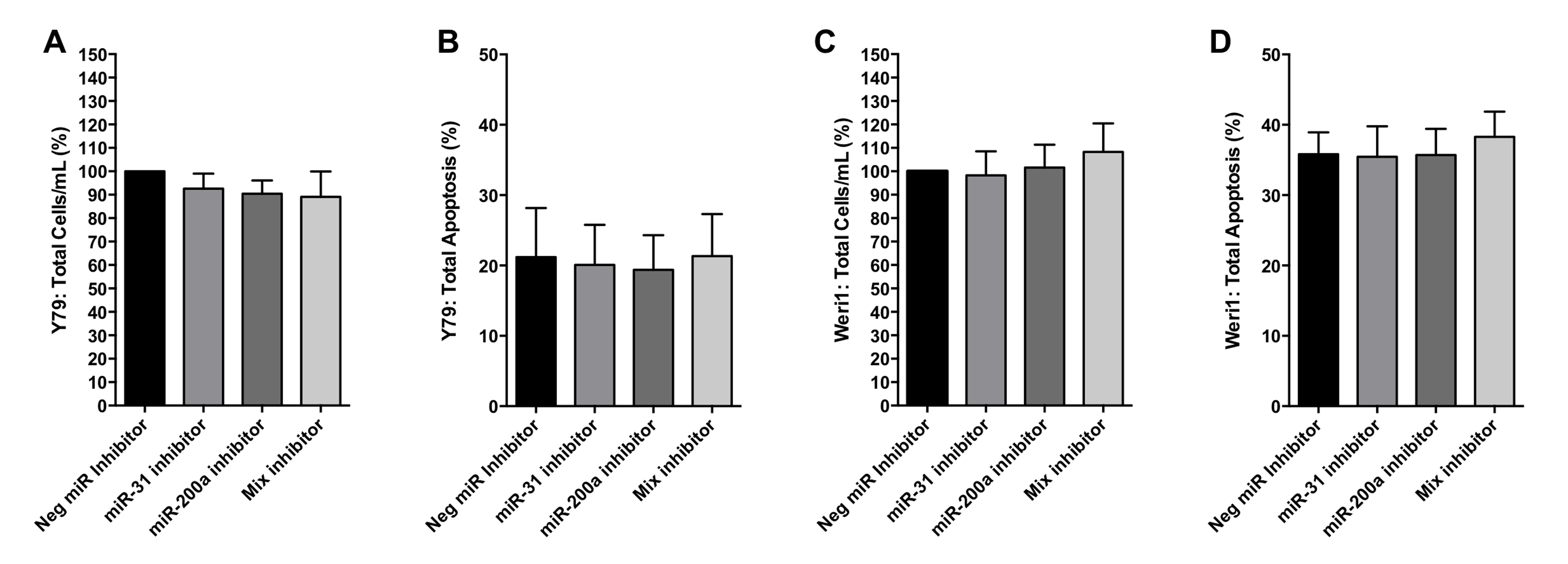

Supplement: S3 Fig — Bar demonstrates percentage in total cells per mL for Y79 (A) and Weri1 (C) at 96 hours post-transfection with indicated miRNA inhibitors for miRNA-31 and/or -200a. Total percent apoptosis (sum of early and late apoptotic percentage of cells) was determined in Y79 (B) and Weri1 (D) cells. Data represents mean and standard deviation from three independent experiments with triplicate samples. (TIF) [file pone.0138366.s004.tif]

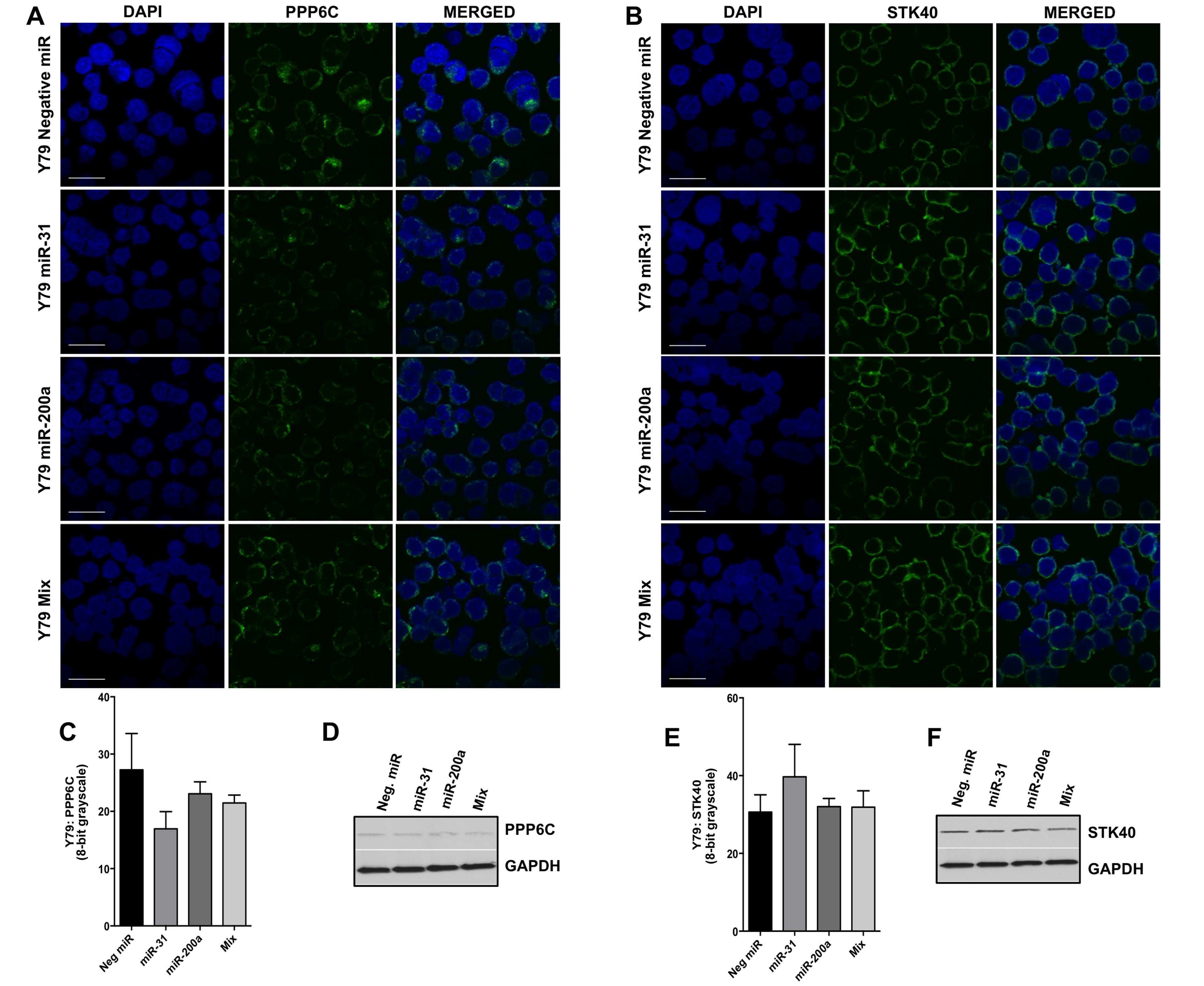

Supplement: S4 Fig — Immunofluorescence staining of PPP6C (A) and STK40 (B) in Y79 cells transfected with a negative miRNA (control), miRNA-31, miRNA-200a, and miR-31/-200a (Mix). Quantification of immunofluorescence of PPP6C (C) and STK40 (E); bar represents mean and standard deviation from two independent experiments with quadruplicate measurements. Western blot analysis from one experiment of PPP6C (D) and STK40 (F) in Y79 cells transfected with a negative miRNA (control), miRNA-31, miRNA-200a, and miR-31/-200a (Mix). Scale bar 20 μm. (TIF) [file pone.0138366.s005.tif]

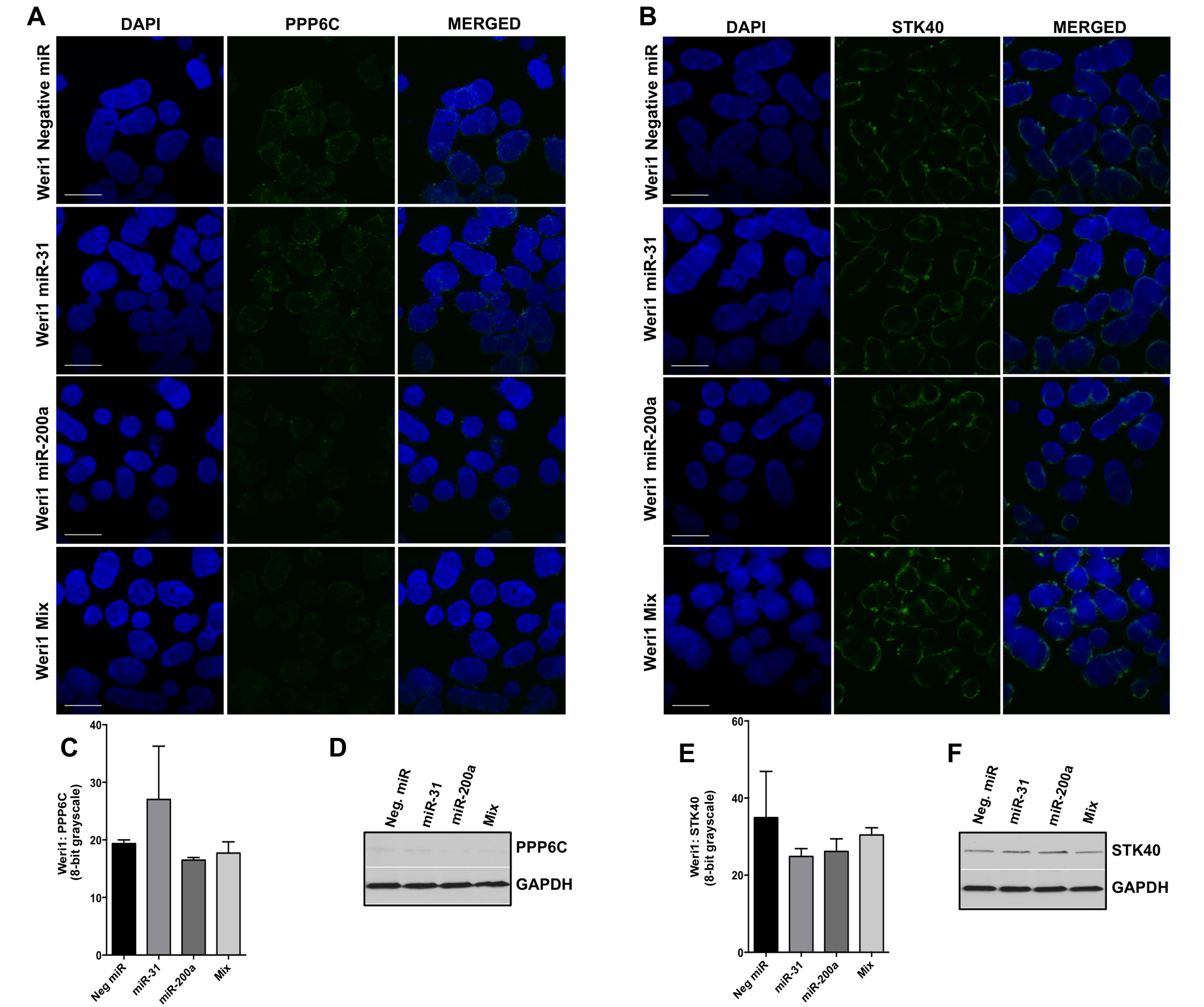

Supplement: S5 Fig — Immunofluorescence staining of PPP6C (A) and STK40 (B) in Weri1 cells transfected with a negative miRNA (control), miRNA-31, miRNA-200a, and miR-31/-200a (Mix). Quantification of immunofluorescence of PPP6C (C) and STK40 (E); bar represents mean and standard deviation from two independent experiments with quadruplicate measurements.Western blot analysis from one experiment of PPP6C (D) and STK40 (F) in Y79 cells transfected with a negative miRNA (control), miRNA-31, miRNA-200a, and miR-31/-200a (Mix). Scale bar 20 μm. (TIF) [file pone.0138366.s006.tif]

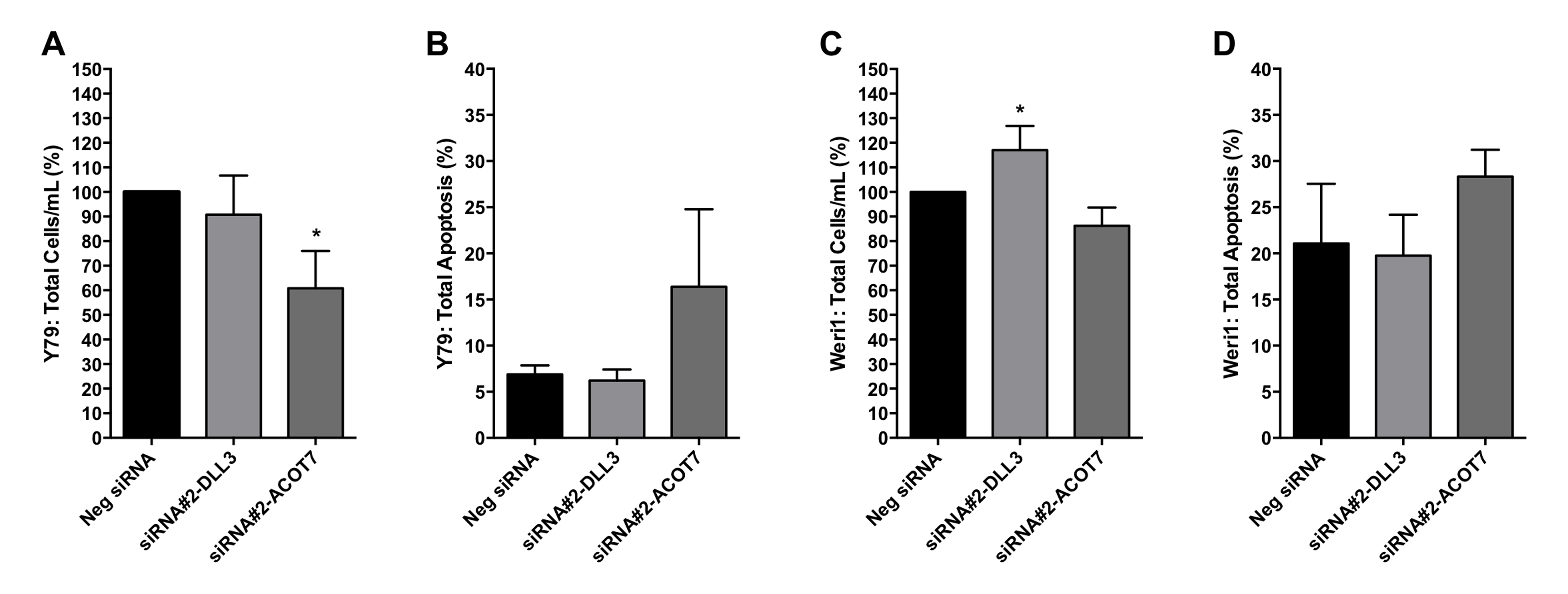

Supplement: S6 Fig — Bar demonstrates percentage difference in total cells per mL for Y79 (A) and Weri1 (C) at 120 hours post-transfection with indicated alternative siRNAs (siRNA#2). Total percent apoptosis (sum of early and late apoptotic percentage of cells) was determined in Y79 (B) and Weri1 (D) cells. Data represents mean and standard deviation from three independent experiments with triplicate samples. * denotes p< 0.05. (TIF) [file pone.0138366.s007.tif]

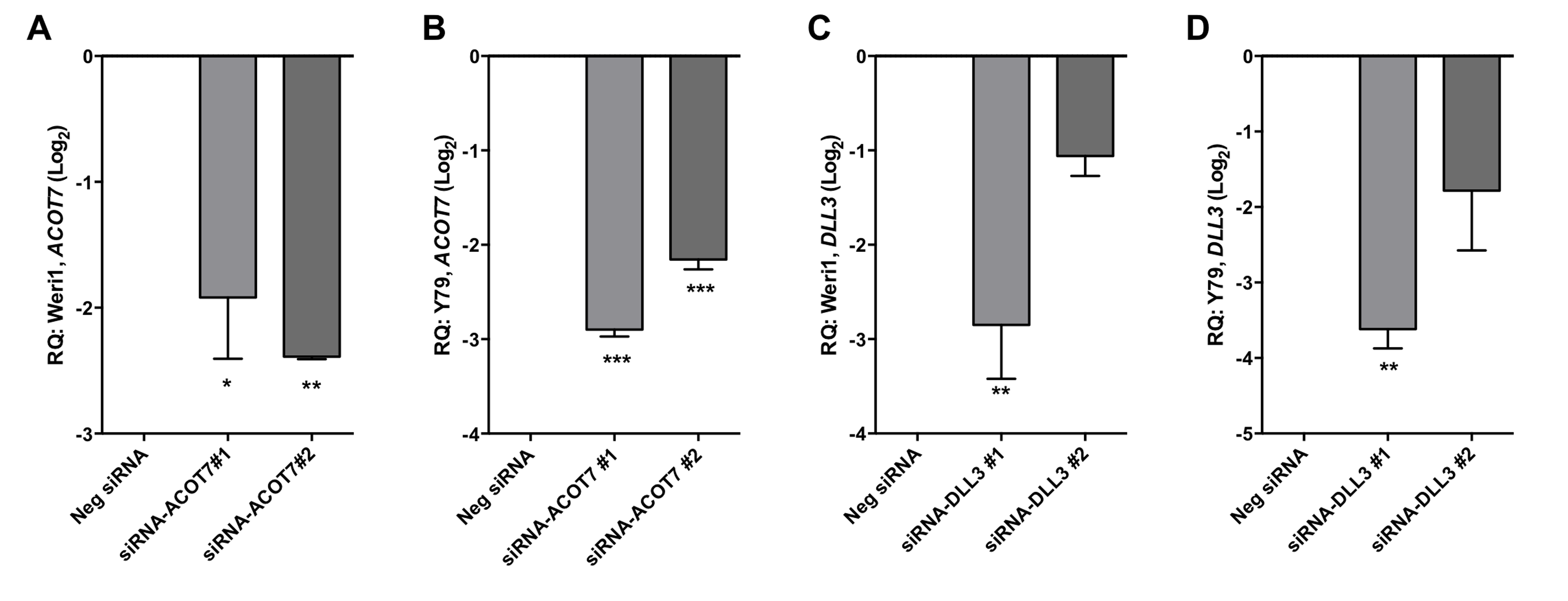

Supplement: S7 Fig — Expression of ACOT7 (A-B) and DLL3 (C-D) as measured by TaqMan qRT-PCR in human retinoblastoma cells (Weri1 and Y79) after transient expression of two distinct siRNAs, as compared to negative control siRNA expressing cells. Data represents mean and standard deviation from two independent experiments. * denotes p<0.05, ** denotes p< 0.01, *** denotes p< 0.001. (TIF) [file pone.0138366.s008.tif]

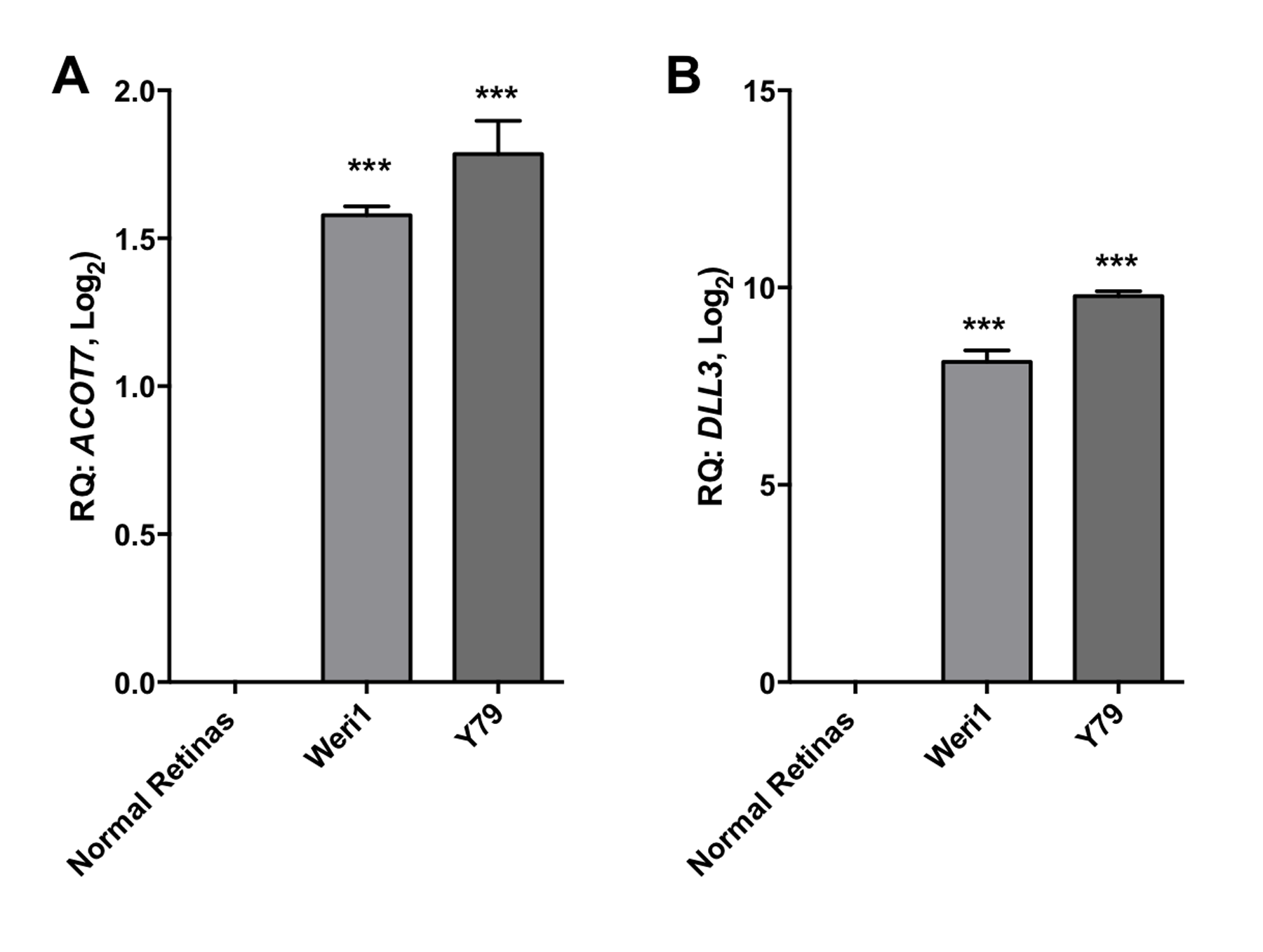

Supplement: S8 Fig — Expression of ACOT7 (A) and DLL3 (B) as measured by TaqMan qRT-PCR in human retinoblastoma cells (Y79 and Weri1) as compared to normal retinas from three individuals. Data represents mean and standard deviation from two experiments with triplicate samples. *** denotes p< 0.0005. (TIF) [file pone.0138366.s009.tif]

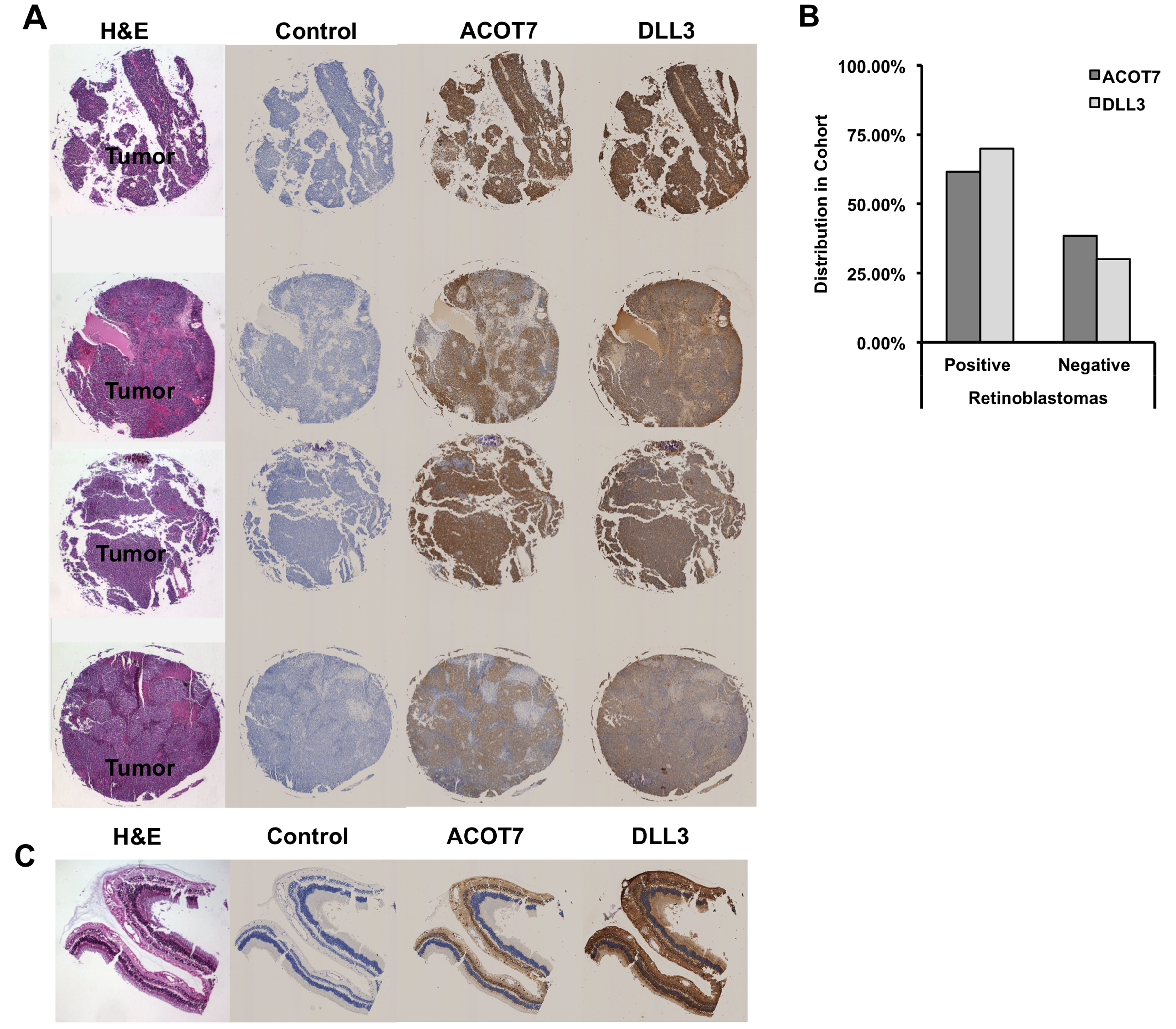

Supplement: S9 Fig — Immunohistochemistry was performed on four retinoblastoma tumor arrays (US Biomax, Catalog BC35111a) for ACOT7 and DLL3. Hematoxylin and eosin staining of retinoblastoma (A) and retinal tissues (C) shows identified tumor (Tumor) and retina, respectively. Control staining served as a negative control (no primary, secondary antibody only, hematoxylin counterstain). (A) Four representative cores from the retinoblastoma tissue array. (B) ACOT7 was detected in 16/26 tumor cores (61.54%) available for analysis. DLL3 was detected in 14/20 tumor cores (70.00%) available for analysis. (C) ACOT7 and DLL3 were detected in 5/5 retinal tissues available for analysis, but shown is one representative specimen. (TIF) [file pone.0138366.s010.tif]

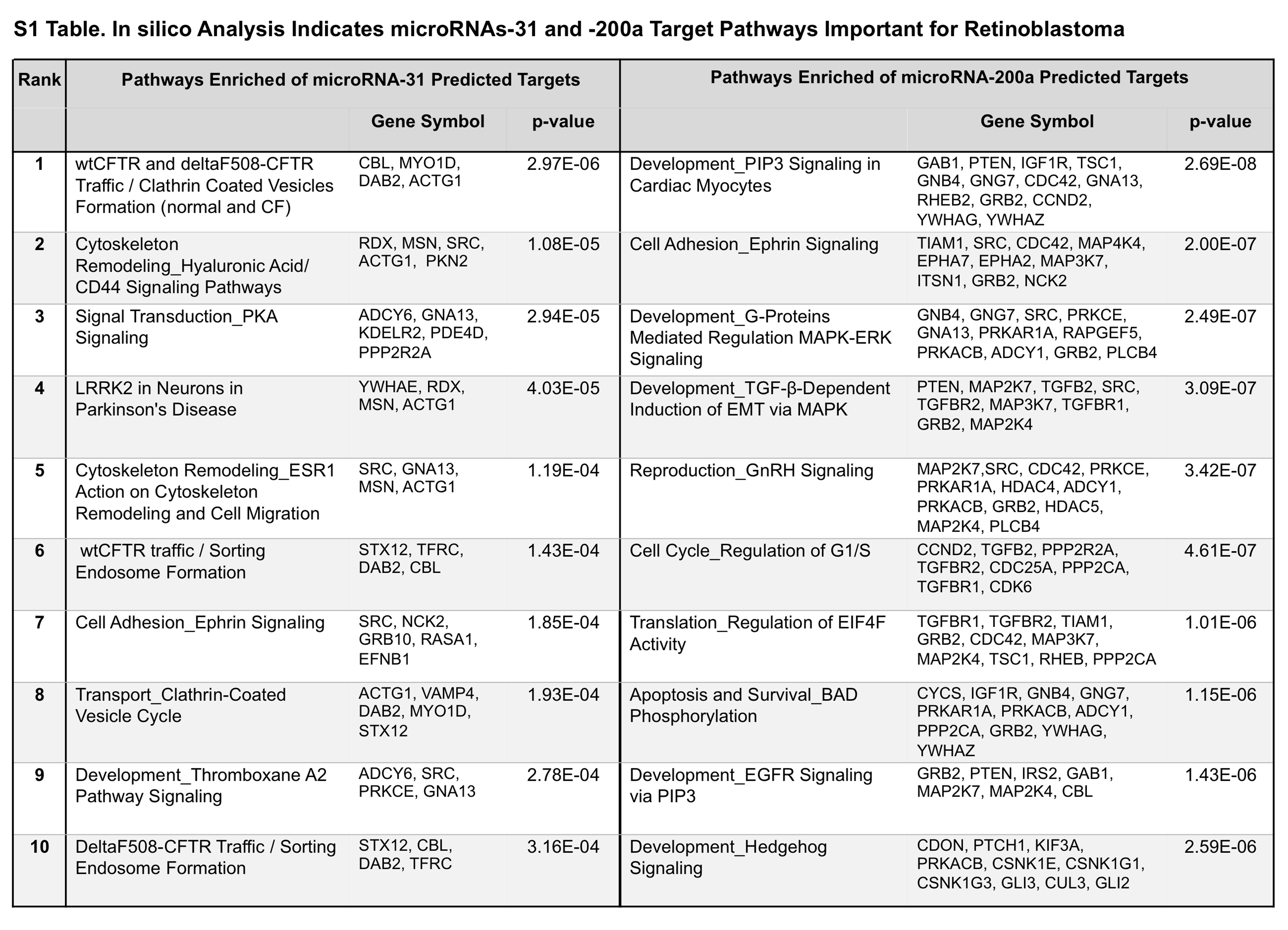

Supplement: S1 Table — Table demonstrates the ten most statistically significant pathways enriched for miRNA-31 or -200a targets. (TIF) [file pone.0138366.s011.tif]
