# Supplementary material for: Novel miRNA-31 and miRNA-200a-Mediated Regulation of Retinoblastoma Proliferation
Source: PLoS One. 2015 Sep 17;10(9):e0138366. doi: 10.1371/journal.pone.0138366 (PMC4574557; doi:10.1371/journal.pone.0138366)
Supplement: S2 Table — (PDF) [file pone.0138366.s012.pdf]

S2 Table

miR-31 Targets	miR-200a Targets
ABLIM1	AADACL1
ACTG1	ABCE1
ADCY6	ABI2
AHCYL1	ABL2
AHSG	ACO1
AK2	ACOT7
ALKBH1	ACPP
ALKBH5	ACVR2A
AMOTL1	ACVR2B
ANKFY1	ADCY1
ANKRD52	ADIPOR2
APBB2	ADPGK
ARHGEF15	ADRB1
ARID1A	AFF1
ARRDC3	AIG1
ATP8A1	AKAP11
ATXN3	AKAP2
B3GNT2	AKAP6
BACH2	ALS2CR4
BAHD1	AMPD2
BAP1	ANKFY1
C11orf41	ANKRD50
C14orf181	ANKRD52
C16orf5	ANP32E
C1orf25	AP1S3
C21orf91	APBB2
C2orf67	APOLD1
CACNB2	APPBP2
CALCR	ARHGAP24
CAMK2D	ARHGAP26
CBL	ARHGEF18
CCDC4	ARID5B
CCNJ	ARL4A
CD28	ARL5B
CDCA7L	ARPC5
CEBPA	ASTN1
CGN	ASXL1
CHD6	ATF6
CHMP7	ATP6V1A
CLASP2	ATP6V1B2
CNNM3	ATP6V1C2

CNP	ATP8A1
COL5A1	ATP8A2
COPS2	ATRN
CORO7	ATXN1
CREG1	ATXN7
CSMD1	ATXN7L1
CTNND2	B3GNT5
DAB2	BACE1
DCBLD2	BACH2
DDX23	BAHD1
DGCR8	BAP1
DICER1	BCAT1
DMD	BCL11B
DOCK1	BCL2L11
DPY19L3	BHLHB2
DPYSL3	BMP1
E2F2	BNC2
EBF3	BRD3
EFNB1	BRD4
EHBP1	BRUNOL6
EHD1	BRWD1
EIF5	C10orf47
EIF5A2	C10orf88
ELAVL1	C10orf97
ELAVL2	C11orf41
EMP1	C11orf61
ENAH	C14orf119
ENTPD1	C14orf135
EPB41L5	C14orf4
FABP2	C14orf43
FAM118A	C14orf83
FAM120C	C18orf1
FAM122A	C18orf19
FAM135B	C18orf25
FAM163A	C1orf173
FAM53B	C1orf21
FAM60A	C21orf91
FEM1C	C3orf67
FGF7	C5orf41
FNDC5	C6orf134
FXR1	C6orf168
FZD3	CALCR
FZD4	CALU

GATAD2B	CANX
GCLM	CARD6
GLT8D3	CBL
GLTSCR1	CBX1
GNA13	CCDC100
GPN1	CCDC4
GRB10	CCDC6
GRIK3	CCDC80
GRPEL2	CCND2
GSTCD	CCNE2
HEATR5A	CCNL2
HELZ	CD47
HIAT1	CDC14A
HIF1AN	CDC25A
HOMER1	CDC25B
HSPC159	CDC2L5
HTR4	CDC42
IHPK1	CDC42EP3
IKZF1	CDC42SE2
IL34	CDK6
INSC	CDON
IQSEC2	CDV3
ISL1	CEP170
JAZF1	CEP350
KANK1	CHD2
KCTD21	CHD9
KDELR2	CHKA
KHDRBS3	CHL1
KIAA0355	CIAO1
KIAA0427	CLASP2
KIAA1024	CLCN5
KIAA1211	CLDN12
KIAA1462	CLIC5
KIAA1576	CLIP2
KIAA1712	CLOCK
KIAA2018	CNOT6L
KIF1B	COQ7
KLF13	CORO1C
KLF3	CPEB3
KLHL3	CPEB4
KRT80	CRLF3
KSR2	CROP
LATS2	CSF3

LEPR	CSNK1E
LHFPL4	CSNK1G1
LMAN2	CSNK1G3
LRRC31	CTBP2
MAP3K1	CTNNA1
MAP3K14	CTNND2
MAP4K5	CUL3
MBOAT2	CUL4B
MDGA1	CXCL12
MED14	CXorf39
METTL2B	CYCS
MLXIP	CYP26B1
MRO	DACT3
MSN	DCP2
MTFR1	DCUN1D3
MYO19	DCX
MYO1D	DDIT4L
NAT13	DDX5
NCK2	DEK
NDRG3	DIP2B
NFAT5	DIS3
NR5A2	DIXDC1
NUFIP2	DLC1
NUMB	DLG3
NXF1	DMWD
ODZ4	DNAJC13
ONECUT2	DOCK4
OSBP2	DOLPP1
OTUD3	DR1
OXSR1	DRD2
PAX5	DUSP3
PAX9	E2F3
PC	EDEM1
PCDHA1	EIF2S3
PCDHA10	EIF5
PCDHA11	ELAVL2
PCDHA12	ELAVL4
PCDHA13	ELF2
PCDHA2	ELL2
PCDHA3	ELMOD1
PCDHA4	ENAH
PCDHA5	EPHA2
PCDHA6	EPHA7

PCDHA7	EPN1
PCDHA8	ERC2
PCDHAC1	ESRRG
PCDHAC2	ETNK1
PDE4D	EVI5L
PDIK1L	EXOC5
PDPR	EXOC7
PDZD4	FAM125B
PEX5	FAM168B
PHC3	FAM46C
PHF21B	FAM84B
PHF8	FAM8A1
PIK3C2A	FAM91A1
PKN2	FAT3
PLXNA2	FBXL19
PLXNA4	FBXL2
POU2F3	FBXL5
PPP1R12B	FBXW2
PPP1R9A	FGFR1OP
PPP2R2A	FKBP5
PPP3CA	FKTN
PPP6C	FLJ12529
PRKCE	FLJ14154
PRSS8	FLJ46838
PTGFRN	FNBP1
RAB27A	FNBP1L
RAB6B	FOXA1
RALGPS1	FOXA2
RAPH1	FOXC1
RASA1	FOXJ1
RBM12	FOXJ3
RBM8A	FOXN2
RBPMS2	FOXN3
RDX	FOXP1
RGAG1	FOXP2
RGS4	FRMD4A
RHBDL3	FRMD6
RHOBTB1	FSD1L
RIMS3	FTO
RNF144A	FUS
RNF150	GAB1
RNF183	GATA6
RSBN1	GCNT2

SALL2	GDF6
SATB2	GGA3
SCG3	GIGYF1
SCN4B	GJC1
SCN9A	GLCCI1
SELT	GLI2
SEPHS1	GLI3
SERF2	GLS
SERINC1	GNA13
SERTAD2	GNB4
SGMS1	GNE
SH2D1A	GNG7
SH3BGRL2	GOLM1
SH3BP1	GOLPH3L
SLC16A2	GPC2
SLC17A8	GPD1
SLC1A2	GPHN
SLC24A3	GPM6B
SLC25A27	GPR109A
SLC2A4	GPR6
SLC43A2	GPRC5C
SLC5A3	GRB2
SLC6A6	GRIN2D
SNN	GRIN3A
SNTB2	GRK6
SNX1	HAS2
SOX11	HDAC4
SPARC	HECTD1
SPOPL	HIC2
SPRY4	HIPK2
SRC	HMG20A
SRGAP3	HMGB1
SS18L1	HMGCS1
SSH1	HNRNPAB
ST3GAL2	HNRNPF
STARD13	HOXA11
STAU2	HOXB5
STK40	HOXC13
STX12	HRB
STX3	HRBL
SUPT16H	HSPC159
SYDE2	IFNAR1
SYT6	IGF1R

TACC1	IGF2
TACC2	IGF2BP2
TAF4	IGSF9B
TBC1D25	IKBKAP
TESK2	IKZF2
TEX261	IPO5
TFRC	IQSEC1
TLN2	IRS2
TM9SF3	ITGA2
TMBIM1	ITSN1
TMED10	JMJD1C
TMEM127	KATNAL1
TMEM145	KBTBD4
TMEM168	KCNJ15
TMEM43	KCNJ2
TMPRSS11F	KCNK5
TNRC6B	KCNN2
TNS1	KCTD2
TTBK1	KCTD20
TTC23	KEAP1
UACA	KHDRBS2
UBE2K	KIAA0408
UBN1	KIAA0574
UCN2	KIAA1012
VAMP4	KIAA1211
VAPB	KIAA1333
VAV3	KIAA1430
VEZT	KIAA1553
VGLL3	KIAA1715
VPS26B	KIAA1826
VPS39	KIAA2018
VWA3A	KIF1B
WDR5	KIF1C
WNK1	KIF3A
XKR6	KLF12
YLPM1	KLF3
YWHAE	KLHL18
ZBTB34	KLHL3
ZC3H12C	KPNA3
ZC3H6	KPNA4
ZDHHC18	LANCL2
ZFHX4	LASS6
ZFP30	LHFP

ZFP36L1	LHX6
ZMAT3	LIFR
ZNF280C	LMO3
ZNF498	LOC162073
ZNF618	LPIN2
ZSCAN18	LPP
	LRRC8A
	LRRTM2
	LYPD6
	LYPLA1
	LYSMD3
	MAF
	MAGED4
	MAGED4B
	MAGI2
	MAL2
	MAP2K4
	MAP2K7
	MAP3K3
	MAP3K7
	MAP4K4
	MAP7D1
	MAPRE2
	MARCH7
	MARK1
	MBNL1
	MBNL3
	MBTD1
	MCOLN3
	MDGA1
	MECP2
	MEGF9
	MGC12966
	MIB1
	MID1
	MIER1
	MLL3
	MN1
	MNX1
	MOBKL2B
	MPPED2
	MSL-1
	MTF2

MTPN
MTSS1
MTTP
MTX3
MYADM
MYBL1
MYH10
MYO1E
MYRIP
MYST4
MYT1L
N4BP1
NAB1
NAP5
NARG2
NCAM1
NCAN
NCK2
NECAB1
NEK6
NFASC
NFYB
NME1
NOPE
NPAL3
NPTX1
NR2C2
NRCAM
NRP1
NRXN1
NUDCD1
NUDT13
NUFIP2
NUMBL
ODZ3
OGT
OLFM1
OLIG3
OSBPL11
OTUD3
OXSR1
P4HB
PAFAH1B2

PAG1
PALM2-AKAP2
PANK3
PAPPA
PAQR9
PAX3
PCDH9
PCGF3
PCM1
PDCD4
PDXP
PDZD2
PEG10
PELI1
PEX5
PGRMC1
PGRMC2
PHC3
PHF21A
PHLPPL
PHYHIPL
PIGW
PIM2
PITPNB
PITX2
PKN2
PLAG1
PLAGL2
PLCB4
PLCXD3
PLXDC1
PLXNA4
POU4F1
PPARA
PPHLN1
PPP1R15B
PPP2CA
PPP2R2A
PPP3R1
PPP3R2
PPT2
PRDM10
PRKACB

PRKAR1A
PRKCE
PSMF1
PTCH1
PTEN
PTP4A1
PTPRD
PTPRG
QKI
QSER1
RAB38
RAB5A
RAG1
RALGPS1
RANBP6
RAPGEF2
RAPGEF5
RARB
RASGEF1A
RBM24
RBM33
RBM47
RBMS1
RCOR3
RDX
RFTN1
RFX1
RGAG1
RGS7BP
RHEB
RHOBTB1
RHPN2
RIMS1
RIPK5
RNF145
RNF185
RNF44
RNFT1
RPH3AL
RRP15
RTN4RL1
RUNX1
RYBP

SCARB2
SCD5
SCHIP1
SCN9A
SDF2
SEC24A
SEMA6A
SENP5
SEPT11
SEPT7
SEPT8
SERF2
SERPINH1
SESN2
SFPQ
SGK269
SHMT2
SHROOM4
SIAH1
SIDT2
SIM2
SIPA1L2
SIRT1
SLAIN2
SLC16A7
SLC17A6
SLC1A1
SLC22A15
SLC23A2
SLC25A3
SLC2A13
SLC30A7
SLC38A1
SLC38A9
SLC5A3
SLC6A17
SLC6A9
SLC7A2
SMARCD1
SMC5
SNAP91
SNF1LK
SNRK

SNRPE
SNTB2
SNX18
SNX27
SNX9
SOBP
SON
SORBS2
SORD
SOX11
SOX17
SOX5
SP4
SPAG9
SPATA2
SPATA5
SPG3A
SPIRE1
SPOCK2
SPRED2
SPRY4
SPRYD3
SRC
SRCAP
SRP72
ST3GAL3
ST3GAL5
ST8SIA5
STAT4
STAT5B
STX16
STX2
STXBP1
SUPT3H
SUPT6H
SV2A
SYT4
SYT6
TACC1
TADA1L
TAF12
TANC2
TAP2

TAPT1
TBR1
TBX4
TCERG1
TCF12
TCF4
TCP11L2
TET1
TET3
TFAP2A
TFAP2B
TFAP2C
TFRC
TGFB2
TGFBR1
TGFBR2
THRB
TIAM1
TM9SF4
TMED5
TMED9
TMEM110
TMEM129
TMEM135
TMEM16F
TMEM170B
TMEM188
TMTC1
TNKS2
TNPO1
TNRC6B
TNS1
TNS3
TP53INP1
TRA2A
TRAM2
TRAPPC2
TRHDE
TRIM2
TRPS1
TSC1
TSHZ3
TTBK2

TTC33
TTLL7
TTPAL
TTYH2
TULP4
TXNIP
UBA6
UBASH3B
UBE2Z
UNC119B
UNC13C
URG4
USP53
USP9X
USP9Y
VDAC3
VGLL3
VPS26B
VSIG8
WAPAL
WDR42A
WDR43
WDR82
WHSC1
WIBG
WIPF1
WNT5A
XKR7
YAF2
YAP1
YPEL5
YTHDC1
YWHAG
YWHAZ
ZBTB34
ZCCHC24
ZCCHC3
ZEB2
ZFAND5
ZFR
ZHX3
ZMPSTE24
ZMYND19

ZNF148
ZNF238
ZNF507
ZNF516
ZNF592
ZNF609
ZNF618
ZNF629
ZNF644
ZNF660
ZNF793
ZNF828
ZYG11B
